# Detecting differential transcript usage in complex diseases with SPIT

**DOI:** 10.1016/j.crmeth.2024.100736

**Published:** 2024-03-19

**Authors:** Beril Erdogdu, Ales Varabyou, Stephanie C. Hicks, Steven L. Salzberg, Mihaela Pertea

**Affiliations:** 1 Center for Computational Biology, Johns Hopkins University, Baltimore, MD, USA; 2 Department of Biomedical Engineering, Johns Hopkins School of Medicine and Whiting School of Engineering, Baltimore, MD, USA; 3 Department of Computer Science, Johns Hopkins University, Baltimore, MD, USA; 4 Department of Biostatistics, Johns Hopkins Bloomberg School of Public Health, Baltimore, MD, USA; 5 Malone Center for Engineering in Healthcare, Johns Hopkins University, Baltimore, MD, USA; 6 Department of Genetic Medicine, Johns Hopkins School of Medicine, Baltimore, MD, USA

**Keywords:** RNA-seq, alternative splicing, DTU, heterogeneity, complex diseases, schizophrenia

## Abstract

Differential transcript usage (DTU) plays a crucial role in determining how gene expression differs among cells, tissues, and developmental stages, contributing to the complexity and diversity of biological systems. In abnormal cells, it can also lead to deficiencies in protein function and underpin disease pathogenesis. Analyzing DTU via RNA sequencing (RNA-seq) data is vital, but the genetic heterogeneity in populations with complex diseases presents an intricate challenge due to diverse causal events and undetermined subtypes. Although the majority of common diseases in humans are categorized as complex, state-of-the-art DTU analysis methods often overlook this heterogeneity in their models. We therefore developed SPIT, a statistical tool that identifies predominant subgroups in transcript usage within a population along with their distinctive sets of DTU events. This study provides comprehensive assessments of SPIT’s methodology and applies it to analyze brain samples from individuals with schizophrenia, revealing previously unreported DTU events in six candidate genes.

## Highlights

• SPIT is a tool for analyzing differential transcript usage in complex populations
• SPIT detects exclusive versus shared DTU events of subgroups
• Robust control of false discovery rates boosts analytical accuracy
• Analyzing schizophrenia RNA-seq data, SPIT discovers DTU events in six key genes

## Motivation

Detecting isoform switch events (differential transcript usage) can provide insights into various disease mechanisms, tissue-specific cell functions, and developmental processes. Frequently, datasets analyzed in these experiments exhibit intricate causal relationships, involving subgroups of patients and cells experiencing distinct events as well as shared events. We sought to develop a tool that considers this structural complexity and identifies differential transcript usage events specific to subgroups within datasets.

## Graphical abstract

**Figure:**
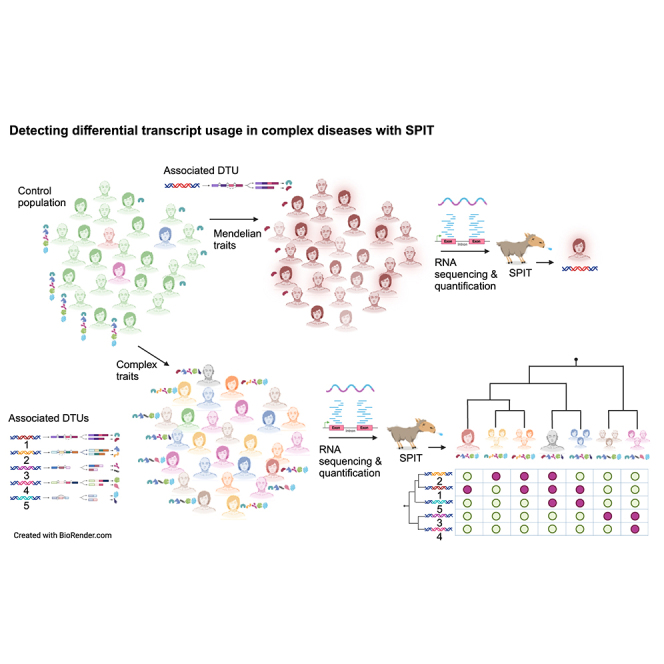

## Teaser

Erdogdu et al. present SPIT, a tool that advances differential transcript usage analysis by navigating population structure and genetic complexity in populations with complex disease. SPIT’s precision in subgroup identification and false discovery rate control makes it a robust tool for revealing intricate transcriptomic variation.

## Introduction

Alternative splicing enables eukaryotic cells to produce a diverse batch of transcripts and, consequently, proteins from a single gene. While for some genes, these distinct transcripts (isoforms) may be used interchangeably, many protein-coding genes have a dominant isoform that is favored in expression across the healthy individuals of a human population.^1^ Predominant expression of alternative isoforms may subject these genes to changes and potential errors in their function.^2^ Differential transcript usage (DTU) analysis is conducted, using RNA sequencing (RNA-seq) data to search for systematic differences in the expression ratios of isoforms that may explain changes in phenotype between cell types, tissues, or populations.^2^^,^^3^

Isoform abundance is often tissue specific, and DTU (also called isoform switching) may result in proteins with distinct functions, which, in turn, may play different roles in the cell.^2^^,^^3^^,^^4^^,^^5^^,^^6^ There is also a growing interest in the effects of DTU in complex human diseases. Instances of DTU have been associated with DNA repair, numerous human cancer types, heart failure, and psychiatric diseases such as autism, schizophrenia, and bipolar disorder.^7^^,^^8^^,^^9^ State-of-the-art DTU analysis tools provide a framework to detect cases where the isoform proportions are consistent within and significantly different between any two groups of samples. However, transcriptomic profiles within populations comprising individuals affected by a complex disease are rarely consistent due to a multiplicity of causal events and disease subgroups; i.e., a cohort of patients diagnosed with the same disease might actually have several distinct underlying genetic disorders.^10^ Therefore, a DTU analysis method that measures and accounts for the structured heterogeneity within complex disease populations is still needed.

We present SPIT, a statistical tool that identifies subgroups within populations at the transcript level and compares their isoform abundance measures. Using both simulated and real RNA-seq data from human heart tissue, we show that SPIT improves specificity rates compared with the state-of-the-art tools with similar sensitivity and detects DTU events exclusive to subgroups as well as DTU events shared among all case samples. Downstream of DTU analysis, SPIT uses detected DTU events to provide insight into potentially hierarchical subgrouping patterns present in complex disease populations using hierarchical clustering.

Within the SPIT algorithm, subgroups with divergent abundance for each transcript are detected using a kernel density estimator, after which the distributions are compared via a nonparametric Mann-Whitney U test. SPIT provides a conservative approximation of the biological and technical variability within datasets with its SPIT-Test module, significantly reducing false discovery rates. Rather than estimating the expression variability per transcript, SPIT-Test samples a null distribution of minimal U statistic p values based on the control group and assumes that, for each transcript, the minimal U statistic p value is drawn from the same underlying distribution when there is no real disease association independent of biological or technical variability.

We applied SPIT to search for DTU events associated with schizophrenia, a psychiatric disorder canonically recognized as a heritable complex disease with an undetermined number of subtypes.^11^^,^^12^^,^^13^ Genetic causes of schizophrenia have long been studied; however, a clear consensus on the level of genetic liability or the acting set of causal events has not been reached to this day. Whole-genome, exome and RNA-seq studies suggest that a wide range of both common and rare genetic variations, including single-nucleotide polymorphisms (SNPs), copy number variations (CNVs), ultra-rare coding variants (URVs), and alternative splicing events, may contribute to the pathogenesis of schizophrenia.^9^^,^^14^^,^^15^^,^^16^ After analyzing RNA-seq data from the dorsolateral prefrontal cortex (DLPFC) of 146 schizophrenia patients and 208 controls, SPIT identified six candidate genes that had statistically significant DTU events associated with schizophrenia. Previously reported disease associations for these candidate genes include neurodegenerative and psychiatric disorders such as Alzheimer’s disease, bipolar disorder, schizophrenia, major depressive disorder, attention deficit hyperactivity disorder, and autism spectrum disorder. No previous report has identified DTU events in any of these genes.

SPIT is open-source software freely available as a PyPI package at https://github.com/berilerdogdu/SPIT. Additionally, a user-friendly Google Colaboratory configuration and step-by-step guide are provided at https://colab.research.google.com/drive/1u3NpleqcAfNz_0EAgO2UHItozd9PsF1w?usp=sharing.

## Results

### A demonstration on simulated data

A DTU event is defined as a significant difference in the proportions of isoforms contributing to the overall expression of a locus between individual or groups of samples. We are particularly interested in cases where there is a clearly dominant isoform in healthy individuals, where DTU can potentially disrupt cellular function and cause anomalies.

**Figure 1:**
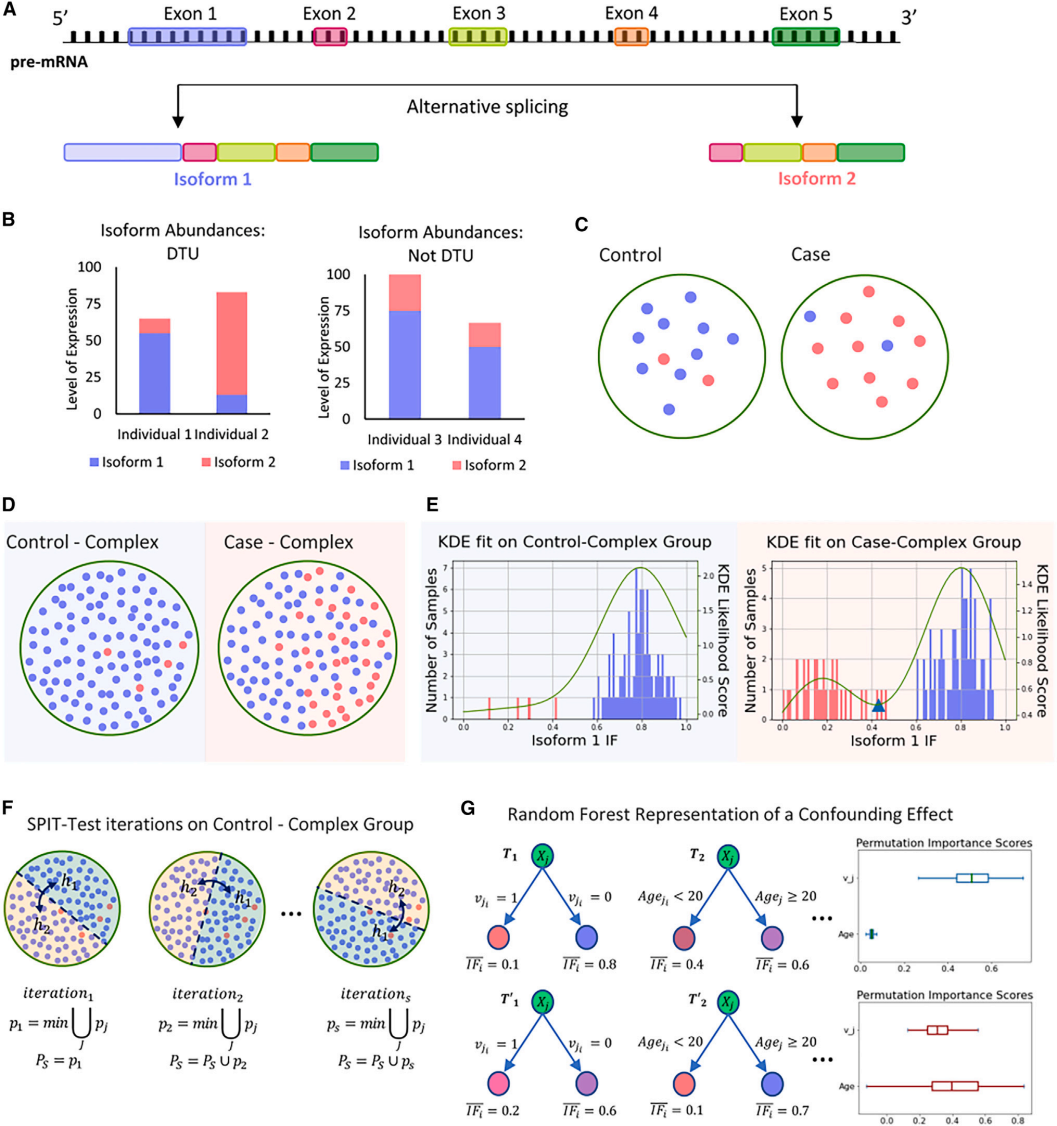
DTU detection demonstration (A) Gene locus going though alternative splicing to produce isoform 1 and isoform 2. (B) Left: isoform abundances in a sample case of DTU between individuals 1 and 2. Right: isoform abundances in a sample case without DTU but with changes in overall expression between individuals 3 and 4. (C) Conventional DTU analysis assumption with no structured heterogeneity in either group. (D) Heterogeneity structure in complex disease samples, where a subset of cases shares the same genetic abnormality (case-complex). (E) Corresponding isoform fraction (IF) distributions and KDE fits for the samples represented in groups control-complex and case-complex. (F) Three SPIT-Test iterations demonstrated with random splits of the control-complex group. (G) Random forest regression representation when there is not a significant confounding effect in the DTU transcript (top) vs. when there is a clear confounding effect by the covariate “age” (bottom). Corresponding permutation importance scores for age and $v_{j}$ are shown on the right. Samples (dots) are color coded based on their dominant isoforms for the locus in (C)–(F). Blue, isoform 1; red, isoform 2.

We describe a modeled DTU case with artificially generated data to exemplify such DTU events and to demonstrate the key steps of the SPIT algorithm. Consider a locus from which two distinct isoforms, isoform 1 and isoform 2, are transcribed, as represented in Figure 1A . Suppose that the protein translated from isoform 1 is a functional protein, whereas isoform 2 is translated into a dysfunctional, aberrant protein. Consequently, the primary expression profile of this locus in a healthy individual is expected to be isoform 1. Figure 1B shows the relative abundances of isoform 1 and isoform 2 for four individuals with varying levels of expression at the locus. The left of Figure 1B demonstrates a clear example of DTU between individual 1 and individual 2, with isoform 1 dominant for individual 1 and isoform 2 dominant for individual 2. The right of Figure 1B illustrates why changes in overall expression at the gene/locus or transcript/isoform level are not sufficient indicators of DTU, as illustrated for the same isoforms in individuals 3 and 4, where overall expression changes, but the relative proportion of the isoforms remains the same.

DTU analysis usually entails comparing two groups of samples rather than individuals. In the interest of brevity, suppose that, for any given individual, either isoform 1 or isoform 2 is significantly dominant for the locus in our model DTU case, and note that each individual is color coded based on their dominant isoform in Figures 1C–1F. Small sample sizes are quite common in RNA-seq experiments,^17^ and Figure 1C represents a typical experiment setup for DTU analysis with 12 samples in each group. For instances where a DTU event between isoform 1 and isoform 2 has a causal link to a disease, Figure 1C depicts the expected scenario for a simple genetic disease where the disease is caused by a single or a small set of genes. In this scenario, one assumes that all or nearly all controls have normal gene expression patterns, while the cases all share a distinct but abnormal gene or transcript expression pattern that has caused them to be placed in the disease cohort.

In contrast, the causal set of genes or events are not expected to be shared among all individuals affected by a complex disorder. The idea that the majority of complex disorders are likely polygenic and that distinct combinations of causal events might lead to similar pathogenesis in different patient groups is widely accepted.^18^ When focusing on a particular causal event, such as the DTU case between isoform 1 and isoform 2, this implies that only a subgroup of patients within the case group is likely to have this event among its causal factors, as depicted in Figure 1D. By segregating this subgroup from the remaining case group, we gain the capability to detect a DTU event that might have otherwise gone unnoticed and to differentiate potential subclusters of the disease group based on shared DTU events.

To do so, we compare the distributions of isoform fractions (IFs) between the two groups, which refers to the proportion of total expression attributed to each isoform. Figure 1E shows the IF levels for isoform 1 in both control-complex and case-complex groups, which is expectedly high for individuals with isoform 1 as the dominant isoform at the locus and low for individuals with isoform 2 as the dominant isoform. By fitting a kernel density estimator (KDE)^19^^,^^20^^,^^21^ on the IF distributions, we can search for bimodality, which, if found, indicates a separation within the groups themselves. Regardless of the number of isoforms or switching events within the same gene, a separation will be detected if the abundance of an isoform shifts for only a subgroup withing the case samples. The right of Figure 1E demonstrates the clear partition of the case-complex subgroups by a global minimum marked with a triangle on the KDE curve. We should note that SPIT does not presuppose the existence of a partition in populations and still detects any shared DTU events in the absence of bimodality.

### Partitioning of subgroups and DTU detection

The transcript counts are transformed into IFs for each sample as follows:

(Equation 1) IFi,j=ti,j∑Gjti,j

where $IF_{i,j}$ is the IF for transcript j in sample i, $t_{i,j}$ is the transcript count for transcript j in sample i, and $G_{j}$ stands for the set of all transcripts that belong to the same gene as transcript j. We fit a KDE with Gaussian kernel^19^^,^^20^^,^^21^ (details on bandwidth selection are described in the STAR Methods section on parameter fitting) on the two vectors of $IF_{I_{c},j}$, where $I_{c}$ stands for the samples in groups c∈{case,control}. If the $IF_{I_{case},j}$ distribution is bimodal, indicating a significant stratification of two subgroups based on the dominance status of transcript j, then we observe this as a global minimum of the KDE (Figure 1E). While we acknowledge the possibility of observing a similar divergence within the control group due to technical or biological variability, our primary objective is to identify subgroups within the case samples for potential associations with disease status. The KDE on the control group is utilized for flagging the most significant candidate DTU genes, as described in the STAR Methods.

There are several advantages to detecting subgroups based on density estimation, the most important of which is the ability to avoid an underlying distribution assumption for the dataset, which can be challenging for RNA-seq-driven data even after multiple normalization steps.^22^ Furthermore, while outlier samples can alter the shape of a KDE, they have a relatively negligible impact on the global minima/maxima as long as appropriate smoothing is applied.^21^ Unlike k-means or hierarchical clustering methods, there is not a hyperparameter that fundamentally affects whether clusters are detected in the data, and the choice of the bandwidth parameter (h) works to our advantage to account for overdispersion by oversmoothing (see STAR Methods section on parameter fitting).

In the presence of a global minimum in the case group at $IF_{i,j}=m_{case}$, we define the left tails of the case and control $IF_{j}$ distributions as the samples that fall to the left of point $m_{case}$ and the right tails as the samples that fall to the right:

(Equation 2) $$\begin{array}{l} l_{case}=\{i\in I_{case}\;|IF_{i,j}\leq m_{case}\}\;\text{and}\;r_{case}=\{i\in I_{case}\;|IF_{i,j}>m_{case}\}\text{,}\\ l_{control}=\{i\in I_{control}\;|IF_{i,j}\leq m_{case}\}\;\text{and}\;r_{control}=\{i\in I_{control}\;|IF_{i,j}>m_{case}\}\text{.} \end{array}$$

To independently search for candidate DTU events in $l_{case}$ and $r_{case}$, the left tails of the case and control $IF_{j}$ distributions are compared internally, as are the right tails, using the non-parametric Mann-Whitney U test; i.e., $\underset{i\in l_{case}}{⋃}IF_{i,j}$ is compared with $\underset{i\in l_{control}}{⋃}IF_{i,j}$, while $\underset{i\in r_{case}}{⋃}IF_{i,j}$ is compared with $\underset{i\in r_{control}}{⋃}IF_{i,j}$. This analysis determines whether the samples in $l_{case}$ could have been drawn from the left-tail control samples with $IF_{i,j}\leq m_{case}$ or whether they exhibit significant differences. Likewise, the same rationale applies for the right tails.

In the absence of a global minimum, a Mann-Whitney U test is conducted between the entire groups of $I_{case}$ and $I_{control}$.

While it is possible for a set of samples to have more than two subgroups for an isoform, such as those with low abundance, moderate abundance (∼0.5), and high abundance, the KDE fitting process tends to oversmooth, making it challenging to observe such subtle distinctions. Attempting to identify fine differences between individuals as a split in distribution is impractical and may result in excessively jittery KDE curves, inflating false discovery rates. In such cases, the nuances among several small subgroups may be overlooked, leading to a general comparison between entire groups of cases and controls. If the shapes of the distributions significantly differ between the case and control groups, then a DTU event will be detected for the entire case group.

### Accounting for inferential uncertainty

DTU analysis is conventionally conducted after transcript quantification, and its accuracy is affected by the uncertainty in mapping reads to transcripts, introducing additional variability in abundance estimates. SPIT adopts a strategy similar to that of the Swish^23^ method by incorporating inferential replicates generated by quantification tools. While SPIT allows for analysis without inferential replicates, if provided by the user, the DTU detection process outlined earlier is reiterated for each replicate to avoid inflated false discovery rates caused by inferential uncertainty. Subsequently, the results from each inferential replicate are combined into a final set of candidate DTU events with a majority vote protocol. For any transcript to be included in this final set, a significant DTU event must be detected in the majority of the inferential replicates.

### Estimating dispersion with SPIT-Test

Although the use of non-parametric statistical tests can help control the false discovery rate (FDR) in differential analyses, the effectiveness of several competing methods is notably diminished when the input data are overdispersed and contains outliers,^24^ a common characteristic of RNA-seq data.^25^ This prevalent phenomenon suggests that we are not capable of precisely estimating dispersion for each individual transcript or gene, in addition to not being able to adequately correct for the vast number of hypotheses being tested. To overcome this challenge, we choose to estimate a single null distribution for the minimal Mann-Whitney U-statistic p values and assume that these observed minimal p values reflect the upper threshold of dispersion in the input dataset.

We want to estimate the lowest expected p values that we might find when there is no real association between a phenotype and changes in isoform abundance. We call this the null distribution $P_{S}$ of the minimal U-statistic p values. To create this estimate ${\hat{P}}_{S}$, SPIT-Test evaluates the control group, in which we assume such an association is absent, although some amount of variation in isoform usage can be observed. As illustrated in Figure 1F, SPIT-Test is an iterative process that randomly splits the control group in half and identifies the greatest difference in IFs across all genes between the two halves. This process gauges the level of random variation (or noise) within the dataset because we assume that disparities between random halves of the control group are not relevant to our search for genuine differences in isoform usage. Later on, the candidate DTU events between the case and control groups are compared, in terms of their significance, with the observed differences between random halves of the control group.

The following steps are performed at each iteration s.

(1) Randomly split the control samples into two sets of equal size, $h_{k,s}$, where k∈{1,2} represents each half for iteration s.
(2) Select a random split point ${ο}_{s}$ to define the left and right tails of each half as

$$l_{h_{1,s}}=\{i\in I_{h_{1,s}}\;|IF_{i,j}\leq o_{s}\}\;\text{and}\;r_{h_{1,s}}=\{i\in I_{h_{1,s}}\;|IF_{i,j}>o_{s}\}\text{,}$$

$$l_{h_{2,s}}=\{i\in I_{h_{2,s}}\;|IF_{i,j}\leq o_{s}\}\;\text{and}\;r_{h_{2,s}}=\{i\in I_{h_{2,s}}\;|IF_{i,j}>o_{s}\}$$

(3) For each transcript j, conduct a Mann-Whitney U test between the sets of $l_{h_{1,s}}$ and $l_{h_{2,s}}$, yielding a Mann-Whitney U-statistic p value, $p_{j_{l,s\;}}$. Similarly, conduct a Mann-Whitney U test between the sets of $r_{h_{1,s}}$ and $r_{h_{2,s}}$, yielding  $p_{j_{r,s}}$.
(4) Assign $p_{j,s}=\min\left(p_{j_{l,s\;}},p_{j_{r,s}}\right)$ to each transcript j for iteration s.
(5) Among the U-statistic p values assigned to all transcripts, store $p_{s}^{\prime }=\min\;{⋃}_{J}p_{j,s}$. To avoid excessive influence from outlier transcripts, we only sample $p^{\prime }$ once from the same transcript throughout all iterations. In other words, in iteration s, we consider transcripts from which $p_{s_{1},\ldots ,s_{n-1}}^{\prime }$ has not been sampled.
(6) ${\hat{P}}_{S}={\hat{P}}_{S}\cup$$p_{s}^{\prime }$.

SPIT-Test estimates dispersion on a global scale, assuming that any transcript could have been subject to the highest observed level of dispersion. Therefore, for an arbitrary transcript j, ${\hat{P}}_{S}$ is considered as an empirical null distribution of the minimal U-statistic p value. This approach emulates the min-P and max-T procedures^26^ and is employed to set a p value threshold, $p_{threshold}^{\prime }$, based on ${\hat{P}}_{S}$ that determines the set of candidate DTU transcripts between case and control samples as

(Equation 3) $$p_{threshold}^{\prime }={\left(\kappa *\left|{\hat{P}}_{S}\right|\right)}^{\text{th}}\;\text{smallest}\;p-\text{value}\;\text{in}\;{\hat{P}}_{S}\text{,}$$

where κ is a user-set parameter. For instance, if κ=0.1 for 1,000 iterations, then the threshold would be the 100th smallest p value. SPIT-Test deviates from a traditional permutation test in its randomization steps 1 and 2 and its exclusion of the case samples due to the potential presence of unknown subgroups. Although κ cannot directly translate into a target family-wise error rate (FWER), we experimentally show that smaller values of κ achieve remarkable control over FWER.

### DTU simulation and evaluation

Simulated RNA-seq reads are conventionally used to evaluate differential analysis tools, as we lack knowledge of ground truth in real data. However, research has consistently shown that simulated reads do not accurately represent the overdispersion levels in real RNA-seq experiments, leading to underestimation of the FDR.^24^^,^^27^ To obtain a more accurate assessment of SPIT’s performance, we make use of both simulated and real RNA-seq data. In these two types of evaluation sets, we compare the true positive rate (TPR) and FDR outcomes of SPIT and the state-of-the-art tools DEXSeq,^28^ DRIMSeq,^29^ satuRn,^30^ edgeR diffSplice,^31^ limma diffSplice,^32^ and Swish.^23^

To improve control over FDR in DEXSeq, DRIMSeq, and satuRn, each analysis was followed by the stage-wise adjustment tool stageR.^33^ In the case of both edgeR diffSplice and limma diffSplice, we applied the Simes adjustment to the obtained p values, aligning with the recommended approach in their documentation for datasets where only a minority of transcripts within a gene exhibit differential usage, which is consistent with the simulation studies incorporated in this evaluation. We evaluate the performance of SPIT with and without the use of inferential replicates when available. Overall, SPIT is the only tool that maintains effective control over FDR across various evaluation test sets while upholding high sensitivity levels. Computation times for each tool on evaluation experiments are summarized in Table S1.

### Evaluation with simulated RNA-seq reads

We borrow the DTU simulation with the largest sample sizes from the “Swimming Downstream” pipeline by Love et al.^34^ as our test dataset with simulated RNA-seq reads (please see the corresponding STAR Methods section for details). This dataset simulates a large number of (>1,500) DTU events in relatively homogeneous populations, resembling the scenario depicted in Figure 1C. While dispersion is incorporated into the transcript expression patterns, there are no subgroups or divergence in the DTU events.

**Figure 2:**
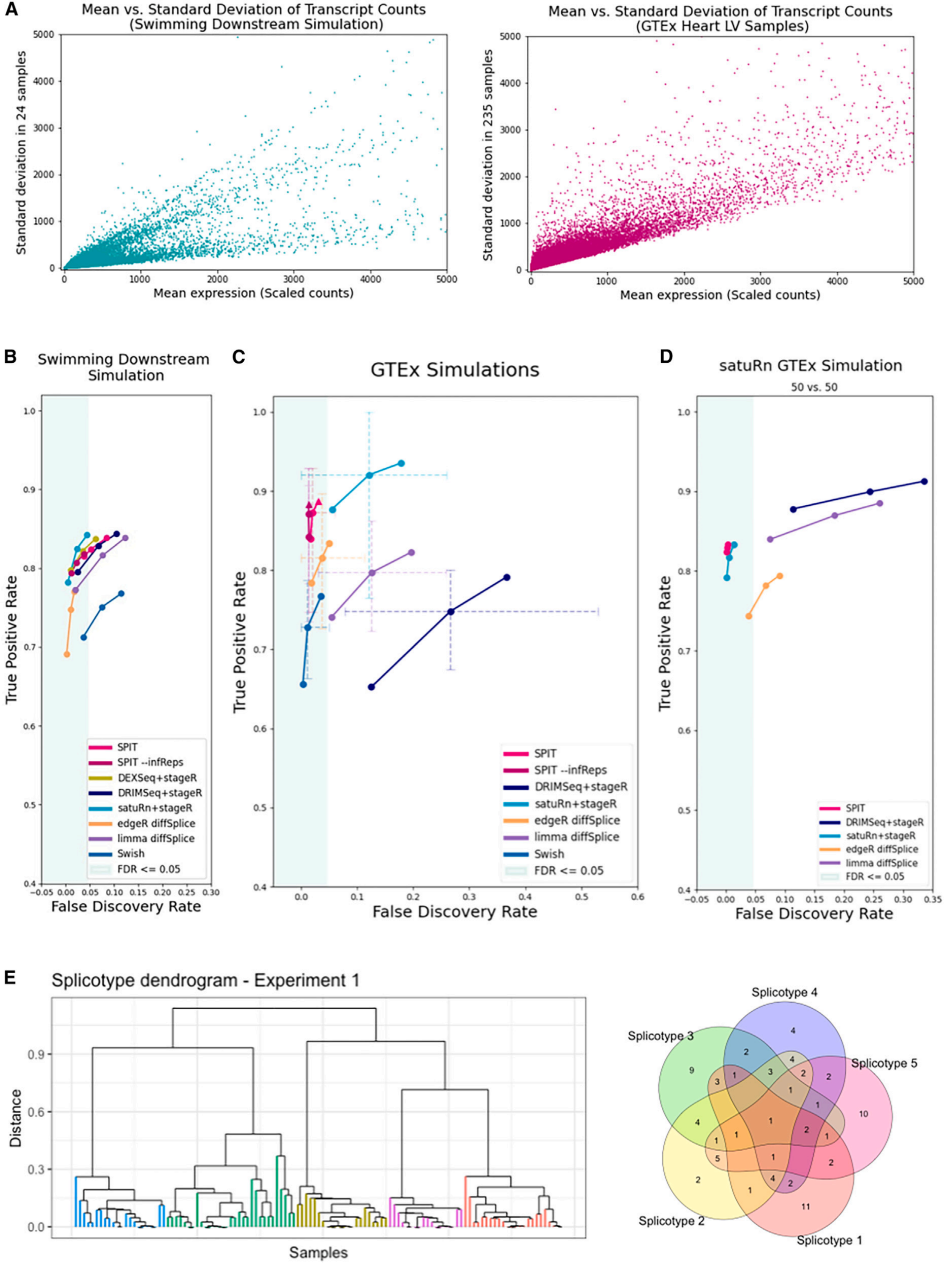
Evaluation (A) Mean vs. standard deviation of the transcript counts are plotted for the “Swimming Downstream” and GTEx experiment samples to represent relative dispersion levels. (B) Gene-level DTU performance evaluation on the “Swimming Downstream” dataset. (C) Gene-level DTU performance evaluation on GTEx experiments. Error bars indicate the minimum and maximum FDR/TPR values obtained. (D) Gene-level DTU performance evaluation on the satuRn (GTEx, 50 vs. 50) simulation. (E) The DTU event sharing Venn diagram for the first experiment in the GTEx simulations (right), and the corresponding final subcluster dendrogram based on the SPIT DTU matrix (left). The subclusters are color coded based on their distinct sets of simulated DTU events (spliceotypes).

The TPR and FDR at the gene level are reported for each tool in Figure 2B, where DEXSeq, DRIMSeq, and satuRn have 3 outcomes corresponding to stageR target overall FDR (OFDR) values 0.01, 0.05, and 0.1. Similarly, for edgeR diffSplice and limma diffSplice, we report 3 outcomes from using target FDR values of 0.01, 0.05, and 0.1 with the Simes adjustment. We used the qvalue^35^ package as outlined in their vignette for Swish to control for local FDR and also provide the results from target FDR values of 0.01,0.05,and0.1. For SPIT, we report 3 outcomes corresponding to setting hyperparameter κ=0.2, 0.4, and 0.6 over 100 iterations. Although the tuning of target OFDR for stageR and κ for SPIT are not directly comparable, lower values of both parameters lead to more conservative behavior, allowing better control over FDR and often yielding decreased TPR.

TPR and FDR outcomes of DEXSeq and DRIMSeq were consistent with the evaluation by Love et al.^34^ Both tools yield high sensitivity levels, while DEXSeq maintained a better control over FDR. edgeR diffSplice, satuRn, and SPIT also exhibit effective FDR control, and using inferential replicates with SPIT reduces false discoveries. Conversely, limma diffSplice, DRIMSeq, and Swish do not display sufficiently low FDR levels. The TPR levels of satuRn, DEXSeq, and SPIT are also more favorable compared with those of edgeR diffSplice and Swish. satuRn achieves the highest TPR level within the FDR ≤0.05 window.

### Evaluation with real RNA-seq reads

#### Genotype-Tissue Expression (GTEx) simulations

To form the basis of our test dataset with real RNA-seq reads, we quantified Illumina reads of 235 normal heart (left ventricle) samples obtained from the GTEx project.^36^ Figure 2A shows the mean-standard deviation plots of the two datasets, revealing a significantly higher level of dispersion in the GTEx dataset compared with the “Swimming Downstream” dataset of simulated RNA-seq reads.

Next, we conducted 20 separate experiments, in each of which we compared random halves of the GTEx dataset after introducing 100 simulated DTU events into one of the halves (please see the corresponding STAR Methods section for details). In an effort to model the expected heterogeneity in a complex disease group, we distributed the 100 DTU events between 5 subgroups in such a way that some DTU events are shared between the subgroups while some are exclusive (see Figure 2E for an example). For the rest of the paper, we will refer to any such subgroup that shares the same DTU events as a “spliceotype” group.

In any random partition of real RNA-seq samples into two groups, it is not certain that there are no actual DTU events beyond the ones we introduced. Therefore, the TPR and FDR measures for the GTEx experiments are only estimates. Our hypothesis for evaluating these experiments was that, if any method consistently detected additional DTU events between random partitions of a healthy sample group, then the discoveries were either noise or else due to biological variance not of interest. Therefore, we present the mean estimated FDR and TPR values of 20 experiments for each DTU tool in Figure 2C, with error bars indicating the minimum and maximum FDR/TPR values obtained. For readability, only the error bars for target FDR levels 0.05 (and κ=0.4 for SPIT) are provided here. Error bars of all target FDR (and κ) values are included in Figure S1.

To run Swish, which requires the use of inferential replicates, we generated pseudo-inferential replicates for each GTEx experiment (for details, please see the relevant section in STAR Methods). Due to its generalized linear model (GLM) fitting step, DEXSeq requires significant computing time for large sample sizes. After running for 168 h on 24 cores and 256 GB RAM, dispersion estimation for the first experiment remained unfinished. Therefore, we do not include DEXSeq in evaluation of this dataset.

In line with the “Swimming Downstream” evaluation, we include results from setting target OFDR values of 0.01, 0.05, and 0.1 for each DTU tool. Because the SPIT pre-filtering process is included in the DTU simulation, we apply each method on the SPIT-prefiltered counts.

In contrast to the TPR and FDR values obtained with the simulated “Swimming Downstream” dataset, every DTU tool yielded a wider range of estimated TPR and FDR values on the GTEx experiments. The FDR estimates increased notably for DRIMSeq, satuRn, and limma diffSplice. We also observe substantially wide error bars for these tools, indicating a large range of performance and lack of consistency across all 20 experiments. This variability could be attributed to the distinct biological differences between the random partitions in each experiment or to the level of heterogeneity introduced in the simulation through varying compositions of DTU events shared between random spliceotypes.

SPIT, edgeR diffSplice and Swish demonstrate effective control over FDR. This is consistent with the “Swimming Downstream” evaluation for SPIT and edgeR diffSplice. Furthermore, we observe relatively narrow error bars for all three of these tools, indicating consistency in performance over 20 experiments. Using inferential replicates with SPIT helps improve FDR control in these experiments as well. The TPR levels of both SPIT and edgeR diffSplice are favorable in this dataset, while SPIT achieves the highest TPR within the FDR ≤0.05 window.

For input datasets with a large number of control samples (n ≥ 32), SPIT offers an optional cross-validation procedure to estimate the optimal value $\kappa^{*}$ based on inferred dispersion, which is detailed in the STAR Methods section on parameter fitting. In Figure 2C, the TPR and FDR obtained using the estimated $\kappa^{*}$ are represented by a triangle, which for this dataset is 0.6. The optimal bandwidth was estimated to be 0.09 through the same procedure. For all other evaluation experiments, the bandwidth was set to 1 as we are not looking for subclusters.

Upon detecting the DTU events for any given dataset, SPIT outputs a binary matrix M of DTU events that marks the presence (1) or absence (0) of a DTU event at the gene level for any sample in the case group relative to the control group. We show that using SPIT’s output matrix M, we are able to cluster the case samples into their separate spliceotype groups based on their shared events by applying hierarchical clustering. The chosen distance metric calculates the proportion of unique events between any two samples relative to the total number of DTU events. As shown in Figure 2E, SPIT perfectly captures the five clusters that were artificially created. Clustering on the first experiment is shown in Figure 2E based on the SPIT output with $\kappa^{*}$; the remaining experiments can be found in Figures S2A–S2S, showing similar results.

#### Null GTEx simulation

For robustness of evaluation, we also generated a null GTEx experiment in which we did not introduce any DTU events into the random halves of the dataset. We ran SPIT, DRIMSeq, edgeR diffSplice, limma diffSplice, and satuRn on the null dataset as described above. SPIT and edgeR diffSplice each reported a single DTU gene, while the other tools reported none. The absence of numerous DTU genes identified by any tool serves as further confirmation that the only reliable signal in the simulated GTEx experiments above is the DTU events introduced the simulation.

#### satuRn GTEx simulations

Both the “Swimming Downstream” dataset and the GTEx experiments we simulated involve at most two transcripts per gene in DTU. In real experiments, it is possible for several transcripts to be involved in switching events. To demonstrate the performance of each DTU detection tool on such events, we borrow the GTEx simulation datasets by Gilis et al.,^37^ which were used in evaluation of the satuRn method. These simulations follow the strategy introduced by Van den Berge et al.^33^ to select the number of transcripts implicated in DTU. For each gene, the number of transcripts are selected from a binomial distribution with n= the number of transcripts of that gene, and p=1/3, allowing for multiple transcripts in DTU. The abundance estimates of the selected transcripts are then swapped.

Three different experiments were generated by Gilis et al.^30^^,^^37^ with sample sizes 50 vs. 50, 20 vs. 20, and 5 vs. 5. For this evaluation, we used the 50 vs. 50 dataset with scaledTPM^38^ counts filtered by the filterByExpr function of edgeR (with default parameters) as provided by Gilis et al.^37^ The sample sizes were too large for running DEXSeq, and inferential replicates were not available to run Swish. We ran the remaining DTU tools using the same approach as in the experiments above without applying any additional filters.

As shown in Figure 2D, DRIMSeq and limma diffSplice demonstrate notably high FDR values coupled with high sensitivity. edgeR controls FDR relatively better but with a lower in TPR than all tools in comparison. SPIT and satuRn are the most effective in controlling FDR while maintaining high sensitivity rates. Their TPR and FDR values closely align, with SPIT exhibiting marginally lower FDR values. The comparison of methods on the 20 vs. 20 dataset is provided in Figure S3. The SPIT algorithm is better suited for analyzing groups of at least 8 samples each, since statistical test approximations will lose power with lower sample sizes, yielding unreliable results. Therefore, the 5 vs. 5 simulation dataset is excluded from our evaluation.

#### Detecting known tissue-dependent DTU events

**Figure 3:**
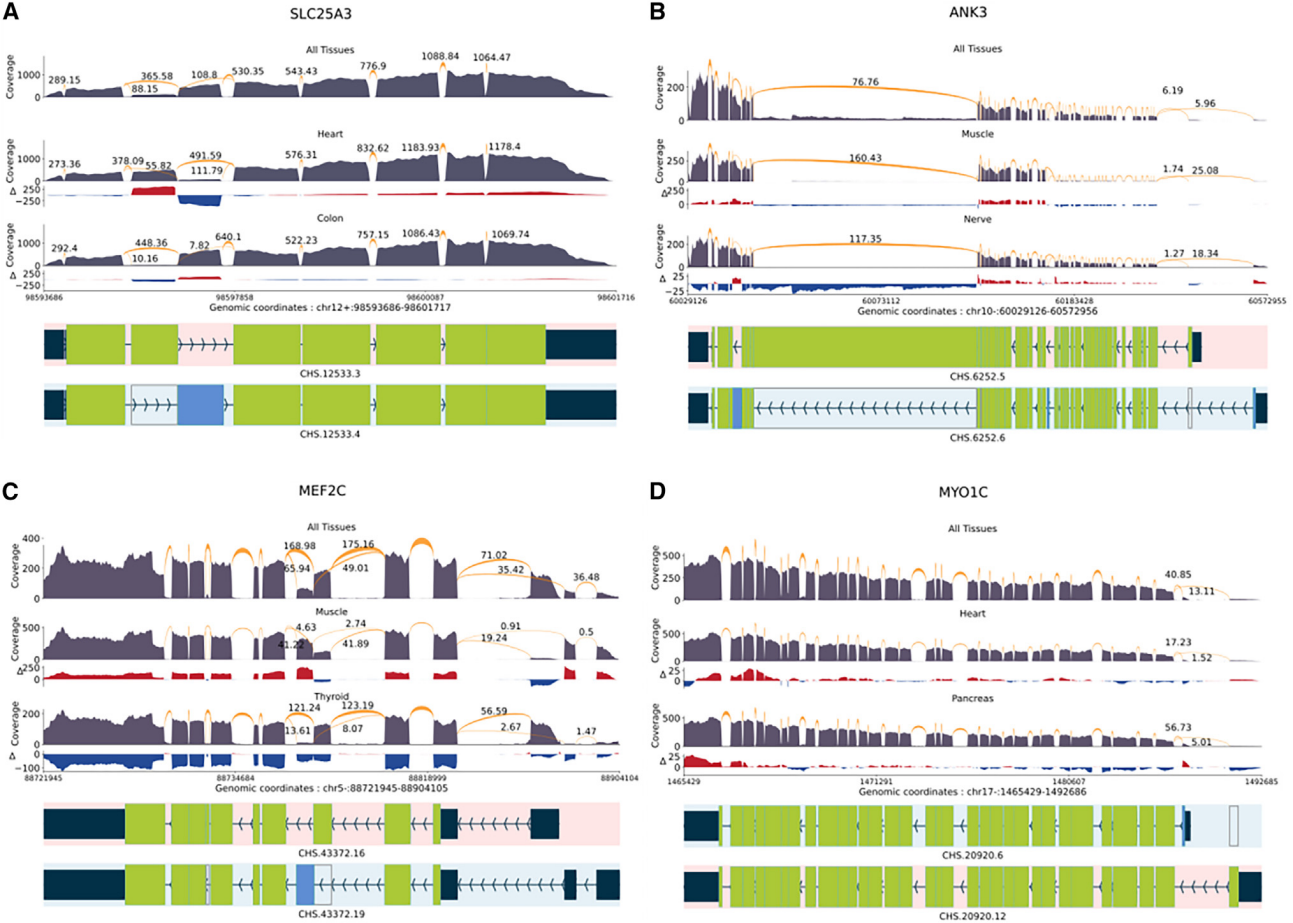
Tissue-dependent DTU events Shown are sashimi plots with normalized coverage and junction values from GTEx samples of the CHESS 3 project. Only the relevant isoforms and junction values are included for readability. The normalized coverage values for each tissue were subtracted from the normalized coverage of the entire GTEx dataset, and the results were illustrated as the Δ track. (A) *SLC25A3* DTU event between heart and colon tissues. (B) *ANK3* DTU event between muscle and nerve tissues. (C) *MEF2C* DTU event between muscle and thyroid tissues. (D) *MTO1C* DTU event between heart and pancreas tissues.

As a positive control experiment, we next investigated a set of four tissue-dependent DTU events that had been previously confirmed individually by various studies and also collectively validated by Reyes and Huber^39^ in 2018. Reyes and Huber^37^ showed that tissue-specific transcript usage is common in humans, and numerous such events are found in each tissue comparison. Our objective was to verify whether SPIT could identify these known examples among the broader pool of detected DTU events. For this analysis, we utilized samples from the GTEx dataset (Table S2) that were aligned as part of the CHESS 3 project.^40^ Figure 3 visually illustrates differentially expressed transcripts between tissues at each locus. All transcriptional landscapes were created using the sashimi plot module in TieBrush after aggregating read alignments from all samples in each tissue. SPIT results on all four DTU events are detailed below. For results from other DTU tools, please refer to STAR Methods and Table S3.

#### SLC25A3

The mitochondrial phosphate transporter gene *SLC25A3* exhibits a phenomenon known as “mutually exclusive exons,”^3^ which refers to the observation that specific exons within the gene are spliced into distinct isoforms, but they are not simultaneously present within the same isoform. We compared 497 samples of heart tissue and 380 samples of colon tissue from the GTEx dataset, and SPIT was able to confirm that one of these isoforms, which is recognized as the primary expression preference in heart and skeletal muscle, is indeed more prevalent in heart tissue samples (Figure 3A).

#### ANK3

Together with two more ankyrin genes, *ANK3* plays a crucial role in generating a diverse array of ankyrin proteins in mammals. Tissue-specific splicing of *ANK3* has been shown previously in skeletal muscle and tibial nerve tissue.^39^^,^^41^ A total number of 480 muscle and 339 nerve tissue samples from GTEx were analyzed using SPIT, confirming the presence of an isoform switch characterized by alternative start sites and distinct patterns of exon splicing (Figure 3B).

#### MEF2C

*MEF2* transcription factors are significant in regulating cell differentiation and expression, and they undergo tissue-specific alternative splicing, adding to their functional diversity. *MEF2C* in humans has two mutually exclusive exons, one of which is shown to be more prevalent in skeletal muscle.^42^ We compared 480 muscle tissue samples from GTEx with 361 thyroid samples using SPIT and were able to detect the isoform switching as a significant DTU event (Figure 3C).

#### MYO1C

*Myosin IC* encodes a protein of the myosin family, which serves multiple cellular functions, including vesicle transportation, transcription, and DNA repair.^43^^,^^44^ The presence of a tissue-dependent transcription start site in *Myosin IC* has been demonstrated, leading to splicing of an alternative first exon,^43^ which SPIT successfully detects upon comparing 497 heart and 199 pancreas samples from GTEx (Figure 3D).

#### Schizophrenia application

After evaluating its performance, we explored the application of SPIT in identifying DTU genes associated with schizophrenia, where we expected a divergence in the causal mechanisms underlying pathogenesis for individual or groups of patients. We obtained RNA-seq samples of postmortem DLPFC tissue from a total of 354 adult brains, which were sequenced by the Lieber Institute for Brain Development.^45^ After applying various quality filtering criteria that are described in detail in the STAR Methods, we selected 146 schizophrenia samples and 208 control samples for comparison in our analysis (Table S4).

**Figure 4:**
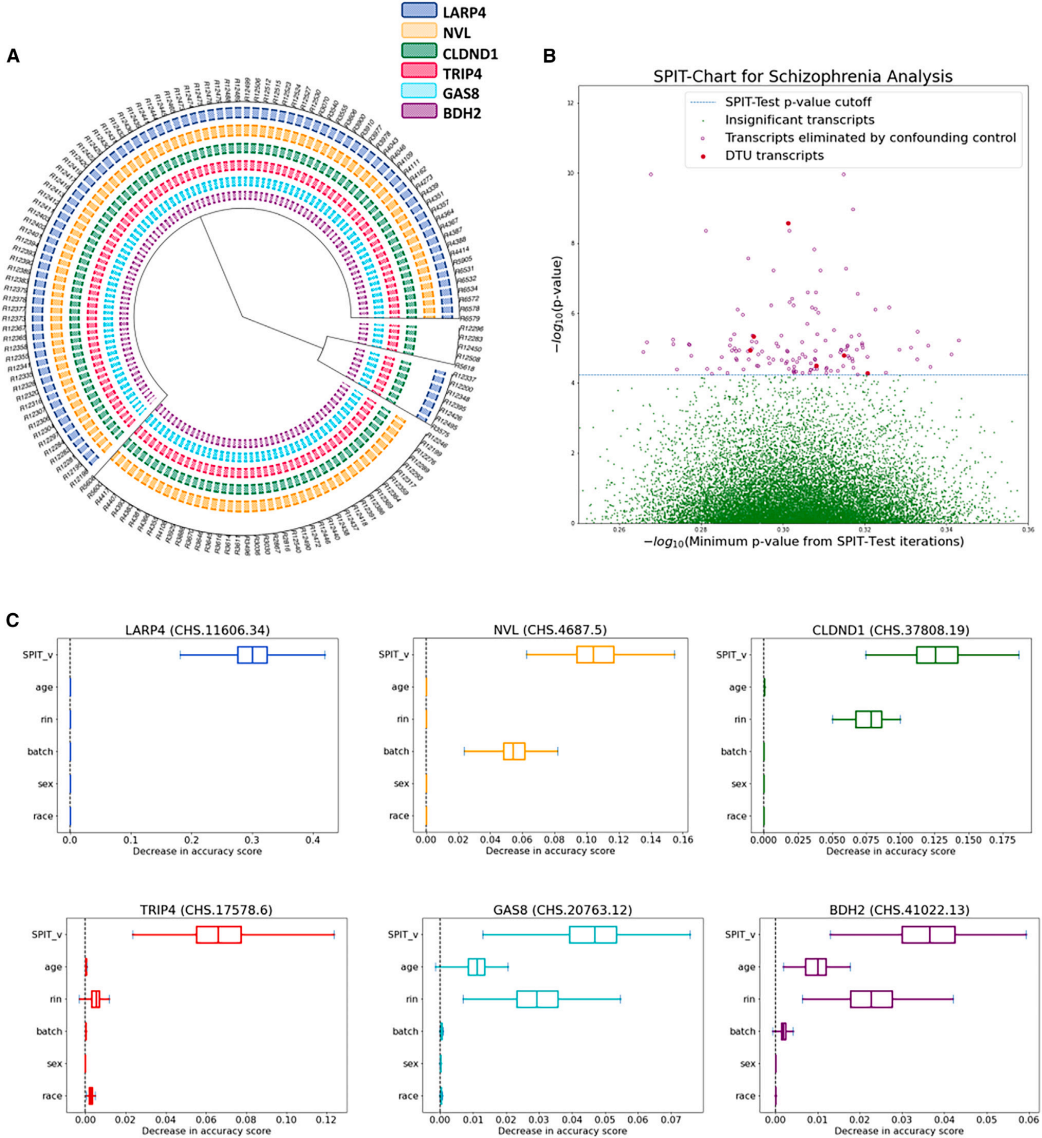
Schizophrenia application (A) Dendrogram representation of hierarchical clustering applied on the SPIT DTU matrix for schizophrenia samples. (B) SPIT-Chart for the schizophrenia analysis. For each transcript that passed the initial filtering steps, the median p value that has been observed through 100 iterations of the SPIT-Test $\left(\text{median}\;\left(\underset{S}{⋃}\;p_{j,s}\right)\right)$ is plotted on the x axis, and the p value observed in the actual comparison of the schizophrenia samples with the controls is plotted on the y axis, both on $-\log_{10}$ scale. (C) Boxplots of permutation importance scores (generated from 100 permutations) of the SPIT output vector and provided covariates for the final 6 DTU genes.

The parameter-fitting process was applied to the control samples, resulting in $\left(h^{*},\kappa^{*}\right)=0.06,0.6$. We took a conservative approach by employing (h,κ)=0.06,0.4. Prior to confounding analysis, SPIT detected 135 potential DTU events between the case and control samples. The binary DTU matrix for these 135 transcripts was then inputted to the confounding control module of SPIT, which is described in the STAR Methods. Covariates considered for all samples included sex, race, age, batch identification, and RNA integrity number (RIN), which highly correlates with RNA degradation.^46^ 129 candidate transcripts were eliminated based on their permutation importance scores, leaving a final set of six DTU transcripts in six genes (Figure 4C). The SPIT-Chart for this analysis (Figure 4A) shows the relationship between the median p values obtained from 100 iterations of SPIT-Test and the p values resulting from comparing control and schizophrenia samples for transcripts.

Among the six candidate genes, four (*BDH2*, *CLDND1*, *GAS8*, and *TRIP4*) displayed DTU events in all schizophrenia samples, while the other two genes (*LARP4* and *NVL*) showed significant DTU events in specific subgroups. Figure 4B depicts the clustering of schizophrenia samples based on identified DTU events, revealing a partitioning into four subgroups in this dataset. We present short descriptions of the functions and associations of the six candidate genes below.

##### *GAS8 (Growth Arrest Specific 8)*

A multitissue study examined SNPs for enrichment of expression quantitative trait loci (eQTLs) across 11 genome-wide association studies (GWASs) focused on schizophrenia and affective disorders (including bipolar disorder, major depressive disorder, autism spectrum disorder, and attention deficit hyperactivity disorder).^47^ The study identified *GAS8* among genes affected by the high-confidence *cis*-eQTLs in multiple brain regions and reported its cross-disorder associations as well as specific associations with bipolar disorder.

##### *NVL (Nuclear VCP Like)*

This gene is a member of the AAA family (ATPases associated with diverse cellular activities) and encodes for two proteins with recognized distinct functions, *NVL1* and *NVL2*,^48^ the latter of which is involved in regulating ribosome biogenesis in eukaryotes.^49^ There is a growing body of evidence suggesting correlations between disrupted ribosome synthesis and aging, as well as neurodegenerative diseases like Alzheimer’s disease and Parkinson’s disease.^50^^,^^51^^,^^52^^,^^53^^,^^54^^,^^55^ In the subset of schizophrenia samples where *NVL* is implicated in DTU, we observed that the *NVL1* isoform was preferred, potentially indicating perturbed ribosomal synthesis (Figure S6).

##### *LARP4 (La Ribonucleoprotein 4)*

The protein encoded by this gene enables RNA-binding activity and plays a critical role in translation regulation.^56^ *LARP4* has been found to show differential expression between the unaffected siblings and first-degree relatives of schizophrenia patients compared with unaffected individuals unrelated to the patients.^57^

##### *BDH2 (3-Hydroxybutyrate Dehydrogenase 2)*

This gene is responsible for encoding a siderophore that plays a crucial role in maintaining iron balance within cells, offering protection against oxidative stress.^58^ Studies have indicated a significant downregulation of *BDH2* in response to inflammation and endoplasmic reticulum (ER) stress.^59^ Disrupted iron homeostasis and ER stress have long been associated with neurodegenerative diseases like Alzheimer’s disease and Huntington’s disease.^60^^,^^61^ Recent studies report *BDH2* to be directly implicated in Alzheimer’s disease progression.^62^

##### *TRIP4 (Thyroid Hormone Receptor Interactor 4)*

The protein encoded by this gene is one of the four components of the activating signal cointegrator 1 (ASC-1) complex. Mutations in ASC-1 components have been described as shared anomalies between the neurodegenerative diseases amyotrophic lateral sclerosis (ALS) and spinal muscular atrophy (SMA).^63^ Mutations in *TRIP4* and *ASCC1*, another component of the ASC-1 complex, are widely recognized as a cause of SMA.^64^^,^^65^

##### *CLDND1 (Claudin Domain Containing 1)*

This gene encodes transmembrane proteins of tight junctions, which play a role in regulating the permeability of brain endothelial cells.^66^ *CLDND1* has been linked to Alzheimer’s disease,^67^ with one study indicating a potential correlation specifically with a subgroup of the condition.^68^

## Discussion

Transcriptomic profiles in populations with complex diseases can exhibit inherent complexity where differentially expressed events are not necessarily shared among all individuals affected by the specific disorder. Consequently, applying the same statistical assumptions for these populations as those used for simple genetic disorders can lead to misleading results in differential analyses. SPIT is built to accommodate and detect structured heterogeneity within populations. Through DTU simulations built on GTEx samples, we show that SPIT not only achieves improved sensitivity and specificity in detecting DTU genes in heterogeneous populations but also successfully captures the specific DTU events for the prevalent subpopulations present.

Our results on the “Swimming Downstream” dataset by Love et al.^34^ also demonstrate that SPIT is equally effective on relatively homogeneous populations and proves to be applicable for diverse scenarios, including simple genetic disorders, tissue-to-tissue comparisons, and other types of DTU studies. SPIT consistently maintains notably low FDRs regardless of the level of dispersion in the datasets.

In addition to simulated experiments, we present four previously confirmed tissue-specific DTU cases that SPIT successfully detected in GTEx samples, as well as six novel DTU associations with schizophrenia. However, to establish any causal link between these six candidate DTU events and schizophrenia, a much more comprehensive investigation is needed, which is beyond the scope of this paper.

### Limitations of the study

SPIT demonstrates optimal performance with larger sample sizes (n ≥ 12), and the use of small sample sizes may lead to a reduction in statistical power, a phenomenon observed in various statistical models. Specifically, with diminished sample sizes, SPIT-Test is susceptible to a loss of range in the null p value distribution. Furthermore, a substantial imbalance between the two groups under comparison may introduce inaccuracies in the results.

It is also crucial to recognize that the DTU associations with schizophrenia reported in this study are exemplars of the application of this method and require further validation. Additional analyses are required to confirm the reported DTU events in diverse settings and populations.

## STAR★Methods

### Key resources table

**Table:** 

REAGENT or RESOURCE	SOURCE	IDENTIFIER
**Deposited data**		

Swimming Downstream simulation	Love et al.^34^	https://zenodo.org/record/1291522
satuRn GTEx simulation	Gilis et al.^30^	https://zenodo.org/records/6826603
20 simulated GTEx experiments	This paper	https://zenodo.org/record/8128846
Quantification files for the GTEx samples used in the tissue-dependent DTU detection	This paper	https://zenodo.org/record/8128945
The RNA-Seq data used in the Schizophrenia analysis	Collado-Torres et al.^45^	http://eqtl.brainseq.org/phase2/

**Software and algorithms**		

SPIT	This paper	https://github.com/berilerdogdu/SPIT https://doi.org/10.5281/zenodo.10695079
*DRIMSeq*	Nowicka and Robinson^29^	https://bioconductor.org/packages/release/bioc/html/DRIMSeq.html
*DEXSeq*	Anders et al.^28^	https://bioconductor.org/packages/release/bioc/html/DEXSeq.html
satuRn	Gilis et al.^30^	https://www.bioconductor.org/packages/devel/bioc/vignettes/satuRn/inst/doc/Vignette.html
*limma diffSplice*	Smyth^32^	https://bioconductor.org/packages/release/bioc/html/limma.html
*edgeR diffSplice*	Chen et al.^31^	https://bioconductor.org/packages/release/bioc/html/edgeR.html
*swish*	Zhu et al.^23^	https://bioconductor.org/packages/release/bioc/vignettes/fishpond/inst/doc/swish.html

### Resource availability

#### Lead contact

Further information and requests for resources should be directed to the lead contact, Beril Erdogdu (berdogd1@jhu.edu).

#### Materials availability

This study did not generate new unique reagents.

#### Data and code availability

• This study utilized publicly available datasets. These and the supporting datasets generated by this study are:
  • The “Swimming Downstream” dataset is uploaded to Zenodo by Love et al.: Quantification files: https://zenodo.org/record/1291522 Scripts and simulation data: https://zenodo.org/record/1410443
  • The satuRn GTEx simulations are uploaded to Zenodo by Gilis et al.*:* https://zenodo.org/records/6826603
  • All 20 of the GTEx simulation experiments are uploaded to Zenodo: https://zenodo.org/record/8128846
  • uantification files and phenotype information for the GTEx samples used in the detection of tissue-dependent DTU events are uploaded to Zenodo: https://zenodo.org/record/8128945
  • The RNA-Seq data used in the Schizophrenia analysis are made available by the Lieber Institute for Brain Development at http://eqtl.brainseq.org/phase2/.
• SPIT is open-source software freely available as a PyPI package and at https://github.com/berilerdogdu/SPIT. Additionally, a user-friendly Google Colaboratory configuration and step-by-step guide are provided at https://colab.research.google.com/drive/1u3NpleqcAfNz_0EAgO2UHItozd9PsF1w?usp=sharing. An archival version of the code is listed in the Key Resources Table.
• Any additional information required to re-analyze the results reported in this paper are available from the lead contact upon request.

### Method details

#### Pre-filtering

The main input SPIT requires is transcript-level count data from an RNA-Seq quantification tool, a mapping file that assigns gene names to each of the transcripts, and any metadata for the samples. Pre-filtering the transcripts before DTU analysis has been shown to improve performance for state-of-the-art tools,^34^^,^^69^ which also holds true for SPIT. The default behavior of SPIT involves the stringent pre-filtering steps listed below which build upon the *DRIMSeq* filtering criteria.

(1) Each transcript must have a Counts per million (CPM) value of at least 1 in at least $n_{small}$ samples, where $n_{small}$ is a user-set parameter that defines the smallest sample size presumed for the subgroups within populations.
(2) Each transcript must have a positive read count in at least a fraction $p_{r}$ of the samples in both the case and control groups, respectively. $p_{r}$ is a user-set parameter and defaults to 00.2.
(3) Each gene must have a read count of at least $g_{c}$ in at least $g_{n}$ samples, where $g_{c}$ and $g_{n}$ are user-set parameters and default to 01.
(4) Each transcript must have an IF value larger than f in at least $n_{small}$ samples, where f is a user-set parameter and defaults to 10..
(5) After the filtering steps above, there must remain at least 2 transcripts for each gene.
(6) The control group must have a consistently dominant isoform for each gene. This criterion is met for a gene when the same isoform of the gene has the largest IF in at least a fraction $p_{d}$ of the control samples, where $p_{d}$ is a user-set parameter and defaults to 0.75.

As is the case for any filtering criteria prior to differential analyses, these steps may inadvertently exclude genuine DTU cases and lower sensitivity. Thus, while these steps are included and recommended in the SPIT pipeline, any or all of them can be excluded from the analysis by the user. Figure S4 outlines the application of this filtering pipeline on the Lieber brain samples discussed in the Results section.

Prefiltering processes in general tend to have a significant impact on the performance of DTU tools.^34^ The effectiveness of filtering criteria also highly depends on the input dataset. In order to observe the effects of applying filters with divergent stringency levels on datasets with varying noise levels, we switched the prefiltering processes applied between our GTEx experiments and the Swimming Downstream Simulation. As discussed above, The GTEx datasets exhibit significantly higher dispersion levels than the Swimming Downstream dataset which is comprised of simulated reads. In the initial analysis, the stringent SPIT prefilters were applied prior to analyzing GTEx experiments for all DTU tools, whereas more lenient filters by *DRIMSeq* were applied on the Swimming Downstream dataset. We now apply the SPIT prefilters on the Swimming Downstream dataset, and the *DRIMSeq* filters on the GTEx experiments (Figure S5).

Switching the prefiltering processes caused an expected harmonious drop in sensitivity levels of all tools in the Swimming Downstream analysis. Interestingly, for the GTEx experiments, the more lenient filters were well-tolerated by the two permutation-test-based methods, SPIT and *Swish*. While the remaining DTU tools displayed a significant jump in their FDR levels with the *DRIMSeq* filter on GTEx experiments, SPIT and *wish* were able to maintain very similar TPR and FDR rates to their performance in the original analysis.

Ultimately, we observe that for DTU analysis, precision and sensitivity are greatly improved by selecting a suitable set of criteria based on the level of dispersion present in the dataset. In real experiments, the user is responsible for determining the level of stringency in their filtering criteria prior to analysis based on their input dataset.

#### Test set with simulated RNA-Seq reads: “Swimming Downstream”

Love et al. simulated DTU events in 1,500 genes by swapping Transcript Per Million (TPM) abundance values between two isoforms. In an additional 1,500 genes, they simulated differential transcript expression (DTE) by altering the abundance value of a single isoform by a fold change between 2 and 6. For these DTE genes, if the differentially expressed transcript is not the only isoform, they were also considered DTU cases as the relative isoform abundances were also expected to change. We include both types of these DTU events as ground truth in our analysis.

Love et al. conducted four experiments with various sample sizes in the case and control groups (n=3vs.3,n=6vs.6,n=9vs.9,n=12vs.12) to evaluate state-of-the-art DTU tools *DEXSeq*, *DRIMSeq*, *RATs*, and *SUPPA2*. They reported that while *SUPPA2* and *RATs* always controlled their FDR, their sensitivity levels remained consistently low across all experiments, hovering around 50%. *DRIMSeq* and *DEXSeq* had considerably higher sensitivity (≥75%) while sometimes exceeding their target FDR. Both *DRIMSeq* and *DEXSeq* demonstrated improved FDR control with larger sample sizes, and 12vs.12 yielded the most favorable TPRs and FDRs.

Based on these findings, we chose to reproduce the “Swimming Downstream” results obtained with *DEXSeq* and *DRIMSeq* on the n=12vs.12 experiment and to evaluate SPIT’s performance on the same dataset. We downloaded the released Salmon^70^ quantification files by Love et al.*,*^71^ and obtained a scaledTPM count matrix for a total number of 203,027 transcripts by running *tximport*. 87,100 of these transcripts had a non-zero scaledTPM counts values in at least one sample.

To facilitate comparisons, Love et al. restrict their evaluation to transcripts and genes that satisfy the *DRIMSeq* filter based on minimum count and abundance estimates, excluding transcripts/genes that do not meet the filter criteria from their set of true positives. We applied the same filters on the input dataset of 203,027 transcripts, and redefined our set of true positives accordingly. We ran each DTU tool on the *DRIMSeq*-filtered count matrix without applying any additional filters. Group 1 was arbitrarily defined as the “control” group in our evaluation.

#### Test set with real RNA-Seq reads: GTEx simulation

To simulate each of the 20 GTEx experiments the following steps were executed.

(1) Randomly divide the 235 GTEx samples into two sets to create case and control groups, $I_{case}$ and $I_{control}$, comprising of 117 and 118 samples, respectively.
(2) Apply the SPIT pre-filter outlined above assuming the randomly assigned $I_{case}$ and $I_{control}$. Note that we skip step 6 of the pre-filtering as it could create an unfair bias in the pre-filtered set of genes toward the DTU genes selected in the next step.
(3) We apply the criteria outlined in step 6 of the pre-filtering process to identify genes with consistently dominant isoforms within the $I_{control}$ group. Out of these genes with dominant isoforms, we randomly select 100 to compose our superset of DTU genes, $D=\left\{d_{1},d_{2},\ldots ,d_{100}\right\}$.
(4) For each spliceotype group (subgroup of samples that share the same DTU events) $\pi_{s},\;s\in \left\{1,2,3,4,5\right\}$, we randomly select 30 DTU genes from set D with replacement to form $D_{\pi_{s}}$. This results in a complex and structured partition within $I_{case}$, where some DTU genes are shared between the five spliceotypes while others are unique to a specific spliceotype.
(5) For a DTU gene $d_{k}\in D_{\pi_{s}}$, let $\alpha_{k}$ be the dominant isoform of $d_{k}$ in $I_{control}$ with $\bar{IF}=u$, and $\beta_{k}$ be the least dominant isoform in $I_{control}$ with mean $\bar{IF}=v$.

We switch the dominance status of $a_{k}$ and $\beta_{k}$ in $I_{case}$ by allowing $IF_{a_{k},i}=v\pm \epsilon$ and $IF_{\beta_{k},i}=u\pm \epsilon$ for all $i\in D_{\pi_{s}}$, where noise parameter ϵ=0.05.

(1) Within all simulated DTU cases, the original transcript counts for $a_{k}$ and $\beta_{k}$ are updated by multiplying the gene counts by $IF_{a_{k},i}$ and $IF_{\beta_{k},i}$, respectively. The gene counts are updated subsequently as the sum of all updated transcript counts, and IF values are calculated once again with Equation 1 so that within each gene IF values add up to 1.

#### Addressing confounding variables

After completing the preliminary DTU analysis, the main output of the SPIT pipeline is a binary vector $v_{j}$ for each transcript indicating the presence (1) or absence (0) of a DTU event in each sample in comparison to the control group. Note that $v_{j}$ carries a 0 for all samples of the control group. Moreover, notice that for the transcripts that SPIT reports as significant DTU events, the $v_{j}$ vector represents a partitioning of all samples, case and control, into two groups with relatively high and low $IF_{j}$ values.

In the presence of a confounding effect, this partition of the high and low $IF_{j}$ values can also be achieved via the confounding variable if included in the experimental design. Based on this assumption, SPIT filters out the DTU events with potential confounding effects using a random-forest-based method.

Given a set of covariates $X{=\{x}_{1},x_{2},\ldots ,x_{k}$}, we define a matrix ${Χ}_{j}$ for every candidate DTU transcript j such that ${{Χ}_{j}}_{i}=\left[{{{v_{j}}_{i},x}_{1}}_{i},{x_{2}}_{i},\ldots ,{x_{k}}_{i}\right]$ for any sample i in either group. We also define a vector $y_{j}$ based on the $IF_{j}$ values such that ${y_{j}}_{i}=I{F_{j}}_{i}$ in the same sample order as in ${Χ}_{j}$.

We then fit a random forest regressor^72^^,^^73^ $\varphi_{j}\left({Χ}_{j}\right)\to y_{j}$ on each candidate DTU transcript. The same number of samples as in the input matrix is bootstrapped for the construction of each tree with maximum tree depth 1, and we minimize the $L_{1}$ loss on the mean $IF_{j}$ in terminal nodes. Notice that with tree-depth 1, our goal is not to precisely predict $I{F_{j}}_{i}$ for samples as much as it is to assess which covariates might be contributing into observable variance in $IF_{j}$ values. We require at least $n_{small}$ number of samples to split the root node. An illustrative case of detecting a confounding effect can be seen in the random forest depicted in Figure 1 g. Building on the modeled demonstration in Figure 1, assume that a candidate DTU event was detected for the subgroup in Case-Complex samples. Supposing one covariate (age) was provided as input, the random forest attempts to regress $IF_{j}$ based on ${{Χ}_{j}}_{i}=\left[{v_{j}}_{i},{\text{age}}_{i}\right]$. On the upper panel, the first tree $T_{1}$ finds the expected effectiveness of vector $v_{j}$ in separating low $IF_{j}$ values, as it was primarily inferred based on $IF_{j}$. A similar effective partition cannot be achieved with the provided covariate age in tree $T_{2}$.

On the lower panel, however, a partition by age in $T_{2}^{\prime }$ demonstrates that age works as well as $v_{j}$ in $T_{1}$, which implies that the identified DTU event cannot be confidently distinguished from a possible confounding effect of the covariate.

With the objective of estimating the importance of each covariate as well as $v_{j}$ in the partitioning of high vs. low $IF_{j}$ samples, we conduct a permutation importance test^73^^,^^74^ on each random forest $\varphi_{j}$. The permutation importance test is based on the coefficient of determination $R_{j}^{2}$ of $\varphi_{j}$, which is a score of how well $IF_{j}$ is predicted in tree leaf nodes.

Let ${\;\varphi }_{j}$ have L leaf nodes $\lambda_{1},\ldots ,\lambda_{l},\ldots ,\lambda_{L}$ with $IF_{j}$ means $\bar{I{F_{j}}_{\lambda_{l}}}$ . Then,

Rj2=1−ujvj, where

$u_{j}=\sum_{l=1}^{L}\sum_{\forall i\in \lambda_{l}}{\left(I{F_{j}}_{i}-\bar{I{F_{j}}_{\lambda_{l}}}\right)}^{2}$, and

$v_{j}=\sum_{I}{\left(I{F_{j}}_{i}-\bar{IF_{j}}\right)}^{2}$.

Once the $R_{j}^{2}$ of $\varphi_{j}$ is calculated on $\varphi_{j}\left({Χ}_{j}\right)\to y_{j}$, one of the covariate columns of the ${Χ}_{j}$ matrix is randomly permuted to form ${Χ}_{j}^{\zeta_{k,\rho }}$, where $\zeta_{k,\rho }$ denotes a random permutation ρ∈Ρ of the covariate $x_{k}$ column. ${R_{j}^{2}}^{\zeta_{x_{k},\rho }}$ is then calculated on $\varphi_{j}\left({Χ}_{j}^{\zeta_{x_{k},\rho }}\right)\to y_{j}$. The importance of covariate $x_{k}$ is then defined as the decrease in score:^74^

(Equation 4) $$\gamma_{x_{k}}=R_{j}^{2}-\;{R_{j}^{2}}^{\zeta_{x_{k},\rho }}\text{.}$$

Although the significance criteria can be changed by the user, in the default settings of SPIT a candidate transcript is only labeled as DTU with the following condition:

(Equation 5) $$Q_{1}{⋃}_{Ρ}\gamma_{v_{j},\rho }\;>\;\max\;{⋃}_{X}Q_{3}\;{⋃}_{Ρ}\gamma_{x_{k},\rho }\text{,}$$

where $Q_{1}$ and $Q_{3}$ refer to the 1^st^ and 3^rd^ quartiles of the permutation importance scores, respectively. The number of permutations for the permutation importance test is a user-set parameter and defaults to 50.

As this confounding-control process is applied subsequent to SPIT-Test on candidate DTU transcripts, strong confounding factors that are disproportionally observed in the control group might still affect the final results. SPIT-Test sets a significance threshold based on the individual differences observed in the control group, and if strong confounding factors are present in the control group and absent in the cases, the SPIT-Test might yield an overly stringent threshold, potentially diminishing sensitivity levels.

#### Parameter-fitting

SPIT has two main hyperparameters that affect its behavior: bandwidth (h) for KDE-fitting, and κ for p-value thresholding. The choice of bandwidth (h) directly determines the level of smoothing in the KDE function, with larger values of h leading to oversmoothed and smaller values leading to undersmoothed IF distributions.^75^ In contrast to the conventional interpretation of an optimal bandwidth, selecting an optimal bandwidth for SPIT does not require achieving the highest possible accuracy in representing the underlying IF histograms. This is due to the fact that overdispersion in RNA-Seq data can lead to overly erratic histograms, which may be identified as multimodal by traditional approaches. Rather, selecting high values of h allows us to reduce the risk of false discoveries by “oversmoothing” the input IF distributions and only detecting only the most significant partitions in the data.

Similar to the choice of bandwidth, the optimal κ value also depends on the level of dispersion present in the input dataset. Smaller values of κ lead to more stringent behavior by setting smaller p-value thresholds for detecting DTU events. To estimate the optimal values of h and κ for each dataset, SPIT implements a parameter-fitting process similar to cross-validation. This involves creating a set of experiments by introducing simulated DTU events into the input control group, following the same approach as used in the GTEx test experiments. Then, different combinations of h and κ values are evaluated based on their accuracy.

Given the set of case samples $I_{case}$ and the set of control samples $I_{control}$, we define a number ($n_{e}$) of experiments, $T=\left\{t_{1},t_{2},\ldots ,t_{n_{e}}\right\}$. To simulate each of the parameter-fitting experiments:

(1) Randomly divide $I_{control}$ into two sets of equal size to create the simulation case and control groups, $I_{case}^{S}$ and $I_{control}^{S}$, respectively.
(2) Apply the SPIT pre-filter outlined above assuming the randomly assigned $I_{case}^{S}$ and $I_{control}^{S}$. As with the GTEx test experiments, we skip step 6 of the pre-filtering process.
(3) We repeat the steps 3–5 of the GTEx test experiment simulation on $I_{case}^{S}$ and $I_{control}^{S}$, where the number of spliceotypes introduced into $I_{case}^{S}$ is a user-set parameter ($n_{g},\text{defaults}\;\text{to}\;5$). For simple genetic disorders and experiments with small sample sizes, $n_{g}$ can be set to 1 as a complex partition within the case group is either not expected or cannot be detected. The noise parameter ϵ can also be set by the user, and defaults to 0.05 as in the GTEx simulation.

In order to estimate the optimal values of h and κ (i.e., $h^{*}$ and $\kappa^{*}$) out of all combinations within search ranges 0.02≤h≤0.20 and κ∈{0.1,0.2,…,1}, we employ a leave-one-out cross-validation (LOOCV) approach on the simulated set of experiments, T. For each step s in $n_{e}$ number of iterations:

(1) Let $T_{\left(s\right)}=T\;\setminus \;t_{s}$. We run SPIT on $T_{\left(s\right)}$ with all $\left(h_{i},\kappa_{j}\right)\;|\;h_{i}\in \left\{0.02,0.03,\ldots ,0.20\right\},\kappa_{j}\in \{0.1,0.2,\ldots ,\;1\}$ to yield estimated F-scores, j.$F_{h_{i},\kappa }$
(2) Select $\left(h_{s}^{*},\kappa_{s}^{*}\right)$ such that j$F_{h_{s}^{*},\kappa_{s}^{*}}=\max\;{⋃}_{I,J}\;F_{h_{i},\kappa }$.
(3) Run SPIT on $t_{s}$ with $\left(h_{s}^{*},\kappa_{s}^{*}\right)$ to get $F_{s}$.

After $n_{e}$ iterations, we obtain a set of optimal hyperparameters and their corresponding F-scores: $\{\left(h_{1}^{*},\kappa_{1}^{*}\right),\left(h_{2}^{*},\kappa_{2}^{*}\right),\ldots ,\left(h_{n_{e}}^{*},\kappa_{n_{e}}^{*}\right)\}$ and ${\{F}_{1},F_{2},\ldots ,F_{n_{e}}\}$. The F-scores are defined as:

(Equation 6) $$F=\frac{2tp}{2tp+tp+fn}\text{,}$$

where tp is the number of true positives, fp is the number of false positives, and fn is the number of false negatives. We select the hyperparameter values with the highest consensus among the iterations as our estimated optimal values $\left(h^{*},\kappa^{*}\right)$. The average F-score ($\bar{F}$) across all iterations can be interpreted as the overall F-score of the SPIT pipeline on the provided dataset, which can help determine if SPIT is an appropriate analysis tool for the dataset. In general, larger sample sizes of the control group (n≥16) are expected to improve accuracy of SPIT test as the U-statistic is nearly normal with n=8vs.8.^76^ Consequently, the parameter-fitting experiments are expected to reveal the best results with control group sizes ≥32.

For the parameter-fitting experiments in this work, we used the default search ranges with $n_{e}=10$ and $n_{g}=5$. $\left(h^{*},\kappa^{*}\right)$ were estimated as (0.09,0.6) for the GTEx simulation experiments, and (0.06,0.6) for the Lieber brain samples. Final $\bar{F}$ across 10 experiments were 0.911 and 0.942, respectively.

SPIT’s parameter-fitting process can be time-consuming and computationally intensive, and it is an optional step. For instance, running 10 experiments (n_e=10) on the 208 control Lieber brain samples took 640 min (10 h 40 min) on a typical personal laptop. However, multithreading is available through GNU parallel.^77^ GNU parallel shares the parameter-fitting experiments between a specified number of threads. As a result, the number of threads GNU parallel will use is limited to the number of experiments, but the time improvement achieved this way is linear. In the case of Lieber brain samples, utilizing 10 threads for 10 experiments reduces this computation time from 640 min to ∼ 1 h. Without parameter-fitting, the default values of (h,κ) are set to the estimated optimal $\left(h^{*},\kappa^{*}\right)$ based on the GTEx dataset (0.09,0.6).

#### Removing outlier effects and tie-correction

Assume that a global minimum was detected in the IF distribution of case samples in order to partition subgroups for an arbitrary transcript, and the left and right tails of the case and control groups were determined as $l_{case}$, $r_{case}$, $l_{control}$, and $r_{control}$.

We define a parameter $n_{small}$, which defines the minimum size for subgroups that can be confidently detected and interpreted in the given dataset. If either or both of the sizes of $l_{control}$ and $r_{control}$ are smaller, they can be expanded to the right and to the left, respectively, until each contains at least $n_{small}$ samples for comparison. Unlike the tails of the control group, $l_{case}$ and $r_{case}$ represent meaningful stratifications within the case group that may have biological implications. Therefore, the group sizes of both $l_{case}$ and $r_{case}$ need to be at least $n_{small}$. Otherwise, the stratification is considered unreliable due to potential influence of outliers. In such cases a Mann-Whitney U test is conducted between the entire groups of $I_{case}$ and $I_{control}$.

Additionally, in order to reduce the impact of insignificant differences between IF values in the Mann-Whitney U test, SPIT rounds all IF values to three decimal points. A random value between −0.0005 and 0.0005 are added to the IF values to break ties. Normal approximation for the U-statistic corrects any remaining ties in the dataset. Although SPIT works well with smaller sample sizes (n≥12) for simple genetic architectures, it requires n≥24 samples for each group for the normal approximation to be reliable in SPIT-Test module. Exact U-statistic p-values are computed for group sizes smaller than 8 when there are no ties.

#### Filtered-CPM threshold

It is worth noting that although $IF_{i,j}$ values are not measures of gene expression, they may still be affected negatively by extremely low gene expression values. For an arbitrary transcript j of gene g, let samples a and b both have $g_{c}=10$, and $t_{a,j}=2$, $t_{b,j}=6$, respectively. As a result we get $IF_{a,j}=0.2$ and $IF_{b,j}=0.6$, which seem to indicate a significant DTU while in reality a difference of 4 in read count is negligible. Therefore, in order to avoid disproportionally inflated differences in $IF_{j}$ values, SPIT has an optional Filtered-Counts per million (CPM) threshold which, for a transcript j of gene g, only considers the samples with CPM ≥10 for g in the Mann-Whitney U test. CPM values for this threshold are calculated on the selected subset of genes that pass the pre-filtering steps above, assigning the total count of these genes as the library size for each sample. This threshold is only used with the real RNA-Seq datasets analyzed in this paper, excluding all simulated experiments and parameter-fitting processes.

#### Flagging DTU genes based on likelihood scores

The KDE-fitting step of SPIT estimates a smoothed distribution for the IF values of each transcript in the case and control groups, which can be exploited to further evaluate candidate DTU events. For an arbitrary DTU event in transcript j between case group $I_{case}$ and control group $I_{control}$, let the estimated kernel densities for $I_{case}$ and $I_{control}$ IF s be $K_{case}^{\prime }$ and $K_{control}^{\prime }$, respectively.

In addition to the Mann-Whitney U statistic between the IF distributions of $I_{case}$ and $I_{control}$, we also calculate the likelihood scores of all $\underset{i\in I_{case}}{⋃}IF_{i,j}$ using density function $K_{control}^{\prime }$, denoted as $L_{j}$. This gives us a measure of the probability of observing the IF values of the case group given the IF distribution of the control samples. Upon collecting the likelihood scores of all transcripts ${⋃}_{J}L_{j}$, we label outlier transcripts using a median absolute deviation (MAD) test with the conventional threshold of 3.5.^78^ As with the U-statistic p-values, presence of subgroups within the case samples results in two separate likelihood scores for a single transcript, in which case the smallest likelihood score gets assigned to the transcript. We assign a significance flag to any candidate DTU gene that has at least one identified outlier transcript.

#### Small samples sizes in SPIT-Test

The Mann-Whitney U statistic approaches a normal distribution for comparisons with sample sizes greater than or equal to 8 vs. 8. With a small control group size (<16), each SPIT-Test iteration will result in at least one random half of the control group with size <8. As the group sizes for comparison decrease, the variability in p-values diminishes, resulting in only the lowest possible p-values, indicative of a full or nearly full separation of ranks between the two groups, being obtained at the end of iterations.

The reduced variability in p-values also introduces a greater dependence on sample size, leading to an imbalance between the p-values obtained through the SPIT-Test and the final p-values derived from the comparison of the actual case and control samples. To handle this, we address the random halves of the control group with a size <8 by imputing random IF values and ensuring that they are completed to match the sizes of the actual control and case groups. For instance, in a specific experiment with control and case group sizes of 8 and 10, respectively, each iteration of the SPIT-Test initially splits the control groups randomly, resulting in groups of 4 vs. 4. Then, these groups are imputed with random IF values to adjust their sizes to 8 vs. 10. The random splitting defined in step 2 of the SPIT-Test iterations is skipped for these samples, conducting the Mann-Whitney U test directly on the imputed groups.

To increase p-value variability for small sample sizes while retaining control over the FDR rate, we also generate the null p-value distribution ${\hat{P}}_{S}$ by randomly selecting a p-value from the 0.01 left tail at each iteration instead of the minimum p-value among all transcripts. For instance, if there are 10,000 transcripts, this corresponds to a randomly selected p-value from the 100 lowest p-values in each iteration.

#### Pseudo-inferential replicates for GTEx experiments

In order to run Swish and SPIT on the simulated GTEx experiments with inferential replicates, the GTEx samples were quantified using Salmon,^70^ generating 30 inferential replicates for each experiment. Each of these inferential replicates were then downscaled to the set of prefiltered transcripts in each experiment, and count estimates were converted to abundance estimates. Finally, for the DTU transcripts in each experiment, the abundance estimates in inferential replicates were replaced with the simulated IF values.

#### Sashimi plots and analysis on tissue-dependent DTU events

To obtain the necessary data, we aggregated the read alignments from all samples in each tissue using TieBrush^79^ and used its module TieCov to extract base-pair and junction coverages.

To manually validate the presence of differentially expressed signals between transcripts at a locus, we constructed sashimi plots for each gene in the evaluation.^79^^,^^80^ These plots, shown for each gene in Figure 3, depict the coverage from each tissue tested for that specific locus. All coverage values obtained using TieCov were normalized using the following formula:

$$\left(\frac{C_{i}}{\sum_{j=0}^{N}C_{j}}\right)\cdot 10^{6}$$

where $C_{i}$ represents the coverage at a given position being normalized, and N is the length of the locus.

To assess differences in the transcriptional landscapes between tissues at each locus, we calculated the change in coverage compared to the average across all GTEx samples (Δ). In Figure 3, the Δ track represents the results obtained by subtracting the normalized coverage values of each tissue from the normalized coverage of the entire GTEx dataset.

In order to run SPIT, all samples were quantified using Salmon^70^ with CHESS 3^41^ as reference annotation. The (h,κ) parameters for SPIT were set as (1,0.6): the bandwidth of 1 ensures that we do not search for subgroups when comparing two tissue types. The $p_{d}$ parameter was set to 0.75 in prefiltering, and the filtered-CPM threshold defined below was employed.

To compare results for the 4 genes with tissue-dependent DTU, we additionally run *DRIMSeq*, *satuRn*, *limma diffSplice*, and *edgeR diffSplice* on the each dataset filtered with the SPIT prefiltering process. All the DTU tools successfully identified the DTU genes being searched for. Since the ground truth of all DTU events is not defined between these tissues, there is no way to compare the robustness of results outside of the queried genes. However, the total number of candidate DTU genes detected by each tool (with target FDR =0.05, and κ=0.6) is different, and provided in Table S3 as a measure of sensitivity and stringency.

For the analysis of genes *ANK3* and *MEF2C*, the empirical p-value correction of *satuRn* fails due to the high number of DTU transcripts. Hence, the raw p-values are used with Benjamini-Hochberg correction^81^ in the results as recommended.

The number of detected DTU events are on the same scale between all tools with the exception of *satuRn*, which yields much smaller sets of DTU genes in experiments with empirical correction. The total compute time of each tool for the experiment with the largest sample sizes (*SLC25A*) is also included in Table S1 serving as a metric for scalability with large datasets.

#### Quantification of the GTEx heart (left ventricle) samples

Transcripts were quantified using Salmon, using the entire genome GRCh38.p14 as a decoy sequence and the reference annotation RefSeq (release 110).^82^ TPM values computed with Salmon were scaled up to library size using the “dtuScaledTPM” conversion from *tximport*.^38^ All downstream analyses used scaled read counts as the unit of expression measurement.

#### Assessment and quantification of Lieber brain samples

The sequencing quality of all brain RNA-Seq samples were assessed with FastQC^83^ and MultiQC,^84^ and outlier samples were excluded from the analysis. Samples with postmortem intervals of ≥60 hours were also excluded. Salmon^70^ was used to quantify all transcripts in the reference annotation CHESS 3^41^ using the entire GRCh38 genome as a decoy sequence. As with the GTEx samples, TPM values computed with Salmon were scaled up to library size using the “scaledTPM” conversion from *tximport*.^38^

As an extra quality control measure, we removed samples with a high proportion of genes exhibiting low expression. To do so, we first calculated the number of genes within each sample with Filtered-CPM ≤10. We then applied the median absolute deviation (MAD) test with a cutoff of 3.5 to remove samples with a significantly higher number of low count genes. The entire pipeline is then rerun on the selected samples, including the pre-filtering steps.

In the SPIT prefiltering process, the $p_{d}$ parameter was set to 0.75, which only includes genes for which the control group predominantly expresses one dominant isoform in the analysis. This is based on the biological plausibility that if a gene has a single dominant isoform in a healthy population, an isoform switching event is more likely to be disruptive. We have also applied the filtered-CPM threshold described above. These strict filtering criteria could result in reduced sensitivity.

### Quantification and statistical analysis

This paper introduces a statistical test, with detailed information available in the preceding section labeled 'method details'.

## Supplemental information

Document S1. Figures S1–S6 and Tables S1–S3

Table S4. Phenotype and background information on the samples used in schizophrenia analysis, related to Figure 4

Document S2. Article plus supplemental information

## References

[1] Ezkurdia I., Rodriguez J.M., Carrillo-de Santa Pau E., Vázquez J., Valencia A., Tress M.L. (2015). Most highly expressed protein-coding genes have a single dominant isoform. J. Proteome Res..

[2] Davuluri R.V., Suzuki Y., Sugano S., Plass C., Huang T.H.M. (2008). The functional consequences of alternative promoter use in mammalian genomes. Trends Genet..

[3] Wang E.T., Sandberg R., Luo S., Khrebtukova I., Zhang L., Mayr C., Kingsmore S.F., Schroth G.P., Burge C.B. (2008). Alternative isoform regulation in human tissue transcriptomes. Nature.

[4] Salomonis N., Schlieve C.R., Pereira L., Wahlquist C., Colas A., Zambon A.C., Vranizan K., Spindler M.J., Pico A.R., Cline M.S. (2010). Alternative splicing regulates mouse embryonic stem cell pluripotency and differentiation. Proc. Natl. Acad. Sci. USA.

[5] de Morrée A., Droog M., Grand Moursel L., Bisschop I.J.M., Impagliazzo A., Frants R.R., Klooster R., van der Maarel S.M. (2012). Self-regulated alternative splicing at the AHNAK locus. Faseb j.

[6] Kellermayer D., Smith J.E., Granzier H. (2017). Novex-3, the tiny titin of muscle. Biophys. Rev..

[7] Vitting-Seerup K., Sandelin A. (2017). The Landscape of Isoform Switches in Human Cancers. Mol. Cancer Res..

[8] Gupta M.P. (2007). Factors controlling cardiac myosin-isoform shift during hypertrophy and heart failure. J. Mol. Cell. Cardiol..

[9] Gandal M.J., Zhang P., Hadjimichael E., Walker R.L., Chen C., Liu S., Won H., van Bakel H., Varghese M., Wang Y. (2018). Transcriptome-wide isoform-level dysregulation in ASD, schizophrenia, and bipolar disorder. Science.

[10] Costa V., Aprile M., Esposito R., Ciccodicola A. (2013). RNA-Seq and human complex diseases: recent accomplishments and future perspectives. Eur. J. Hum. Genet..

[11] Arnedo J., Svrakic D.M., Del Val C., Romero-Zaliz R., Hernández-Cuervo H., Fanous A.H., Pato M.T., Pato C.N., de Erausquin G.A., Molecular Genetics of Schizophrenia Consortium (2015). Uncovering the hidden risk architecture of the schizophrenias: confirmation in three independent genome-wide association studies. Am. J. Psychiatry.

[12] Liu Z., Palaniyappan L., Wu X., Zhang K., Du J., Zhao Q., Xie C., Tang Y., Su W., Wei Y. (2021). Resolving heterogeneity in schizophrenia through a novel systems approach to brain structure: individualized structural covariance network analysis. Mol. Psychiatry.

[13] Tsuang M.T., Lyons M.J., Faraone S.V. (1990). Heterogeneity of Schizophrenia: Conceptual Models and Analytic Strategies. Br. J. Psychiatry.

[14] Ripke S., Neale B.M., Corvin A., Walters J.T.R., Farh K.-H., Holmans P.A., Lee P., Bulik-Sullivan B., Collier D.A., Huang H. (2014). Biological insights from 108 schizophrenia-associated genetic loci. Nature.

[15] Marshall C.R., Howrigan D.P., Merico D., Thiruvahindrapuram B., Wu W., Greer D.S., Antaki D., Shetty A., Holmans P.A., Pinto D. (2017). Contribution of copy number variants to schizophrenia from a genome-wide study of 41,321 subjects. Nat. Genet..

[16] Singh T., Neale B.M., Daly M.J. (2020). Exome Sequencing Identifies Rare Coding Variants in 10 Genes Which Confer Substantial Risk for Schizophrenia. medRxiv.

[17] Soneson C., Delorenzi M. (2013). A comparison of methods for differential expression analysis of RNA-seq data. BMC Bioinf..

[18] Wray N.R., Lee S.H., Mehta D., Vinkhuyzen A.A.E., Dudbridge F., Middeldorp C.M. (2014). Research review: Polygenic methods and their application to psychiatric traits. J. Child Psychol. Psychiatry.

[19] Murray R. (1956). Remarks on Some Nonparametric Estimates of a Density Function. Ann. Math. Stat..

[20] Emanuel P. (1962). On Estimation of a Probability Density Function and Mode. Ann. Math. Stat..

[21] Bernard S.W. (1986).

[22] Hawinkel S., Rayner J.C.W., Bijnens L., Thas O. (2020). Sequence count data are poorly fit by the negative binomial distribution. PLoS One.

[23] Zhu A., Srivastava A., Ibrahim J.G., Patro R., Love M.I. (2019). Nonparametric expression analysis using inferential replicate counts. Nucleic Acids Res..

[24] Li J., Tibshirani R. (2013). Finding consistent patterns: a nonparametric approach for identifying differential expression in RNA-Seq data. Stat. Methods Med. Res..

[25] Robinson M.D., McCarthy D.J., Smyth G.K. (2010). edgeR: a Bioconductor package for differential expression analysis of digital gene expression data. Bioinformatics.

[26] Westfall P.H., Young S.S. (1993).

[27] Varabyou A., Salzberg S.L., Pertea M. (2021). Effects of transcriptional noise on estimates of gene and transcript expression in RNA sequencing experiments. Genome Res..

[28] Anders S., Reyes A., Huber W. (2012). Detecting differential usage of exons from RNA-seq data. Genome Res..

[29] Nowicka M., Robinson M.D. (2016). DRIMSeq: a Dirichlet-multinomial framework for multivariate count outcomes in genomics. F1000Res..

[30] Gilis J., Vitting-Seerup K., Van den Berge K., Clement L. (2021). satuRn: Scalable analysis of differential transcript usage for bulk and single-cell RNA-sequencing applications. F1000Res..

[31] Chen Y., McCarthy D., Ritchie M., Robinson M., Smyth G., Hall E. (2020).

[32] Smyth G.K. (2004). Linear models and empirical bayes methods for assessing differential expression in microarray experiments. Stat. Appl. Genet. Mol. Biol..

[33] Van den Berge K., Soneson C., Robinson M.D., Clement L. (2017). stageR: a general stage-wise method for controlling the gene-level false discovery rate in differential expression and differential transcript usage. Genome Biol..

[34] Love M.I., Soneson C., Patro R. (2018). Swimming downstream: statistical analysis of differential transcript usage following Salmon quantification. F1000Res..

[35] Storey J.D., Tibshirani R. (2003). Statistical significance for genomewide studies. Proc. Natl. Acad. Sci. USA.

[36] (2015). Human genomics. The Genotype-Tissue Expression (GTEx) pilot analysis: multitissue gene regulation in humans. Science.

[37] Gilis J., Vitting-Seerup K., Van den Berge K., Clement L. (2022).

[38] Soneson C., Love M.I., Robinson M.D. (2015). Differential analyses for RNA-seq: transcript-level estimates improve gene-level inferences. F1000Res..

[39] Reyes A., Huber W. (2018). Alternative start and termination sites of transcription drive most transcript isoform differences across human tissues. Nucleic Acids Res..

[40] Varabyou A., Sommer M.J., Erdogdu B., Shinder I., Minkin I., Chao K.-H., Park S., Heinz J., Pockrandt C., Shumate A. (2022). CHESS 3: an improved, comprehensive catalog of human genes and transcripts based on large-scale expression data, phylogenetic analysis, and protein structure. bioRxiv.

[41] Hopitzan A.A., Baines A.J., Ludosky M.-A., Recouvreur M., Kordeli E. (2005). Ankyrin-G in skeletal muscle: Tissue-specific alternative splicing contributes to the complexity of the sarcolemmal cytoskeleton. Exp. Cell Res..

[42] Hakim N.H.A., Kounishi T., Alam A.H.M.K., Tsukahara T., Suzuki H. (2010). Alternative splicing of Mef2c promoted by Fox-1 during neural differentiation in P19 cells. Gene Cell..

[43] Sielski N.L., Ihnatovych I., Hagen J.J., Hofmann W.A. (2014). Tissue specific expression of myosin IC isoforms. BMC Cell Biol..

[44] Cook A.W., Gough R.E., Toseland C.P. (2020). Nuclear myosins – roles for molecular transporters and anchors. J. Cell Sci..

[45] Collado-Torres L., Burke E.E., Peterson A., Shin J., Straub R.E., Rajpurohit A., Semick S.A., Ulrich W.S., Price A.J., BrainSeq Consortium (2019). Regional Heterogeneity in Gene Expression, Regulation, and Coherence in the Frontal Cortex and Hippocampus across Development and Schizophrenia. Neuron.

[46] Gallego Romero I., Pai A.A., Tung J., Gilad Y. (2014). RNA-seq: impact of RNA degradation on transcript quantification. BMC Biol..

[47] Bhalala O.G., Nath A.P., Inouye M., Sibley C.R., UK Brain Expression Consortium (2018). Identification of expression quantitative trait loci associated with schizophrenia and affective disorders in normal brain tissue. PLoS Genet..

[48] Germain-Lee E.L., Obie C., Valle D. (1997). NVL: A New Member of the AAA Family of ATPases Localized to the Nucleus. Genomics.

[49] Nagahama M., Hara Y., Seki A., Yamazoe T., Kawate Y., Shinohara T., Hatsuzawa K., Tani K., Tagaya M. (2004). NVL2 is a nucleolar AAA-ATPase that interacts with ribosomal protein L5 through its nucleolar localization sequence. Mol. Biol. Cell.

[50] Jiao L., Liu Y., Yu X.-Y., Pan X., Zhang Y., Tu J., Song Y.-H., Li Y. (2023). Ribosome biogenesis in disease: new players and therapeutic targets. Signal Transduct. Target. Ther..

[51] Stein K.C., Morales-Polanco F., van der Lienden J., Rainbolt T.K., Frydman J. (2022). Ageing exacerbates ribosome pausing to disrupt cotranslational proteostasis. Nature.

[52] Flach J., Bakker S.T., Mohrin M., Conroy P.C., Pietras E.M., Reynaud D., Alvarez S., Diolaiti M.E., Ugarte F., Forsberg E.C. (2014). Replication stress is a potent driver of functional decline in ageing haematopoietic stem cells. Nature.

[53] Ding Q., Markesbery W.R., Chen Q., Li F., Keller J.N. (2005). Ribosome Dysfunction Is an Early Event in Alzheimer's Disease. J. Neurosci..

[54] Ding Q., Zhu H., Zhang B., Soriano A., Burns R., Markesbery W.R. (2012). Increased 5S rRNA Oxidation in Alzheimer's Disease. J. Alzheimers Dis..

[55] Healy-Stoffel M., Ahmad S.O., Stanford J.A., Levant B. (2013). Altered nucleolar morphology in substantia nigra dopamine neurons following 6-hydroxydopamine lesion in rats. Neurosci. Lett..

[56] Yang R., Gaidamakov S.A., Xie J., Lee J., Martino L., Kozlov G., Crawford A.K., Russo A.N., Conte M.R., Gehring K., Maraia R.J. (2011). La-related protein 4 binds poly(A), interacts with the poly(A)-binding protein MLLE domain via a variant PAM2w motif, and can promote mRNA stability. Mol. Cell Biol..

[57] Glatt S.J., Stone W.S., Nossova N., Liew C.C., Seidman L.J., Tsuang M.T. (2011). Similarities and differences in peripheral blood gene-expression signatures of individuals with schizophrenia and their first-degree biological relatives. Am. J. Med. Genet. B Neuropsychiatr. Genet..

[58] Devireddy L.R., Hart D.O., Goetz D.H., Green M.R. (2010). A mammalian siderophore synthesized by an enzyme with a bacterial homolog involved in enterobactin production. Cell.

[59] Zughaier S.M., Stauffer B.B., McCarty N.A. (2014). Inflammation and ER stress downregulate BDH2 expression and dysregulate intracellular iron in macrophages. J. Immunol. Res..

[60] Vidal R., Caballero B., Couve A., Hetz C. (2011). Converging pathways in the occurrence of endoplasmic reticulum (ER) stress in Huntington's disease. Curr. Mol. Med..

[61] Matus S., Glimcher L.H., Hetz C. (2011). Protein folding stress in neurodegenerative diseases: a glimpse into the ER. Curr. Opin. Cell Biol..

[62] Bai B., Wang X., Li Y., Chen P.-C., Yu K., Dey K.K., Yarbro J.M., Han X., Lutz B.M., Rao S. (2020). Deep Multilayer Brain Proteomics Identifies Molecular Networks in Alzheimer’s Disease Progression. Neuron.

[63] Chi B., O'Connell J.D., Iocolano A.D., Coady J.A., Yu Y., Gangopadhyay J., Gygi S.P., Reed R. (2018). The neurodegenerative diseases ALS and SMA are linked at the molecular level via the ASC-1 complex. Nucleic Acids Res..

[64] Knierim E., Hirata H., Wolf N.I., Morales-Gonzalez S., Schottmann G., Tanaka Y., Rudnik-Schöneborn S., Orgeur M., Zerres K., Vogt S. (2016). Mutations in Subunits of the Activating Signal Cointegrator 1 Complex Are Associated with Prenatal Spinal Muscular Atrophy and Congenital Bone Fractures. Am. J. Hum. Genet..

[65] Oliveira J., Martins M., Pinto Leite R., Sousa M., Santos R. (2017). The new neuromuscular disease related with defects in the ASC-1 complex: report of a second case confirms ASCC1 involvement. Clin. Genet..

[66] Shima A., Matsuoka H., Yamaoka A., Michihara A. (2021). Transcription of CLDND1 in human brain endothelial cells is regulated by the myeloid zinc finger 1. Clin. Exp. Pharmacol. Physiol..

[67] Patel H., Dobson R.J.B., Newhouse S.J. (2019). A Meta-Analysis of Alzheimer's Disease Brain Transcriptomic Data. J. Alzheimers Dis..

[68] Neff R.A., Wang M., Vatansever S., Guo L., Ming C., Wang Q., Wang E., Horgusluoglu-Moloch E., Song W.-m., Li A. (2021). Molecular subtyping of Alzheimer’s disease using RNA sequencing data reveals novel mechanisms and targets. Sci. Adv..

[69] Soneson C., Matthes K.L., Nowicka M., Law C.W., Robinson M.D. (2016). Isoform prefiltering improves performance of count-based methods for analysis of differential transcript usage. Genome Biol..

[70] Patro R., Duggal G., Love M.I., Irizarry R.A., Kingsford C. (2017). Salmon provides fast and bias-aware quantification of transcript expression. Nat. Methods.

[71] Love M.I. (2018).

[72] Breiman L. (2001). Random Forests. Mach. Learn..

[73] Pedregosa F., Varoquaux G., Gramfort A., Michel V., Thirion B., Grisel O., Blondel M., Prettenhofer P., Weiss R., Dubourg V. (2011). Scikit-learn: Machine Learning in Python. J. Mach. Learn. Res..

[74] Altmann A., Toloşi L., Sander O., Lengauer T. (2010). Permutation importance: a corrected feature importance measure. Bioinformatics.

[75] Jones M.C., Marron J.S., Sheather S.J. (1996). A Brief Survey of Bandwidth Selection for Density Estimation. J. Am. Stat. Assoc..

[76] Mann H.B., Whitney D.R. (1947). On a Test of Whether one of Two Random Variables is Stochastically Larger than the Other. Ann. Math. Statist..

[77] Tange O. (2018).

[78] Iglewicz B., Hoaglin D.C. (1993).

[79] Varabyou A., Pertea G., Pockrandt C., Pertea M. (2021). TieBrush: an efficient method for aggregating and summarizing mapped reads across large datasets. Bioinformatics.

[80] Katz Y., Wang E.T., Silterra J., Schwartz S., Wong B., Thorvaldsdóttir H., Robinson J.T., Mesirov J.P., Airoldi E.M., Burge C.B. (2015). Quantitative visualization of alternative exon expression from RNA-seq data. Bioinformatics.

[81] Benjamini Y., Hochberg Y. (1995). Controlling the False Discovery Rate: A Practical and Powerful Approach to Multiple Testing. J. Roy. Stat. Soc. B.

[82] O'Leary N.A., Wright M.W., Brister J.R., Ciufo S., Haddad D., McVeigh R., Rajput B., Robbertse B., Smith-White B., Ako-Adjei D. (2016). Reference sequence (RefSeq) database at NCBI: current status, taxonomic expansion, and functional annotation. Nucleic Acids Res..

[83] Andrews S. (2010). Babraham Bioinformatics.

[84] Ewels P., Magnusson M., Lundin S., Käller M. (2016). MultiQC: summarize analysis results for multiple tools and samples in a single report. Bioinformatics.

