# Supplementary material for: Detecting differential transcript usage in complex diseases with SPIT
Source: Cell Rep Methods. 2024 Mar 19;4(3):100736. doi: 10.1016/j.crmeth.2024.100736 (PMC10985272; doi:10.1016/j.crmeth.2024.100736)
Supplement: Document S1. Figures S1–S6 and Tables S1–S3 [file mmc1.pdf]

Cell Reports Methods, Volume *4*

Supplemental information

Detecting differential transcript usage in complex diseases with SPIT

Beril Erdogdu, Ales Varabyou, Stephanie C. Hicks, Steven L. Salzberg, and Mihaela Pertea

Supplementary Figure 1: Evaluation including error bars for all points, related to Figure 2

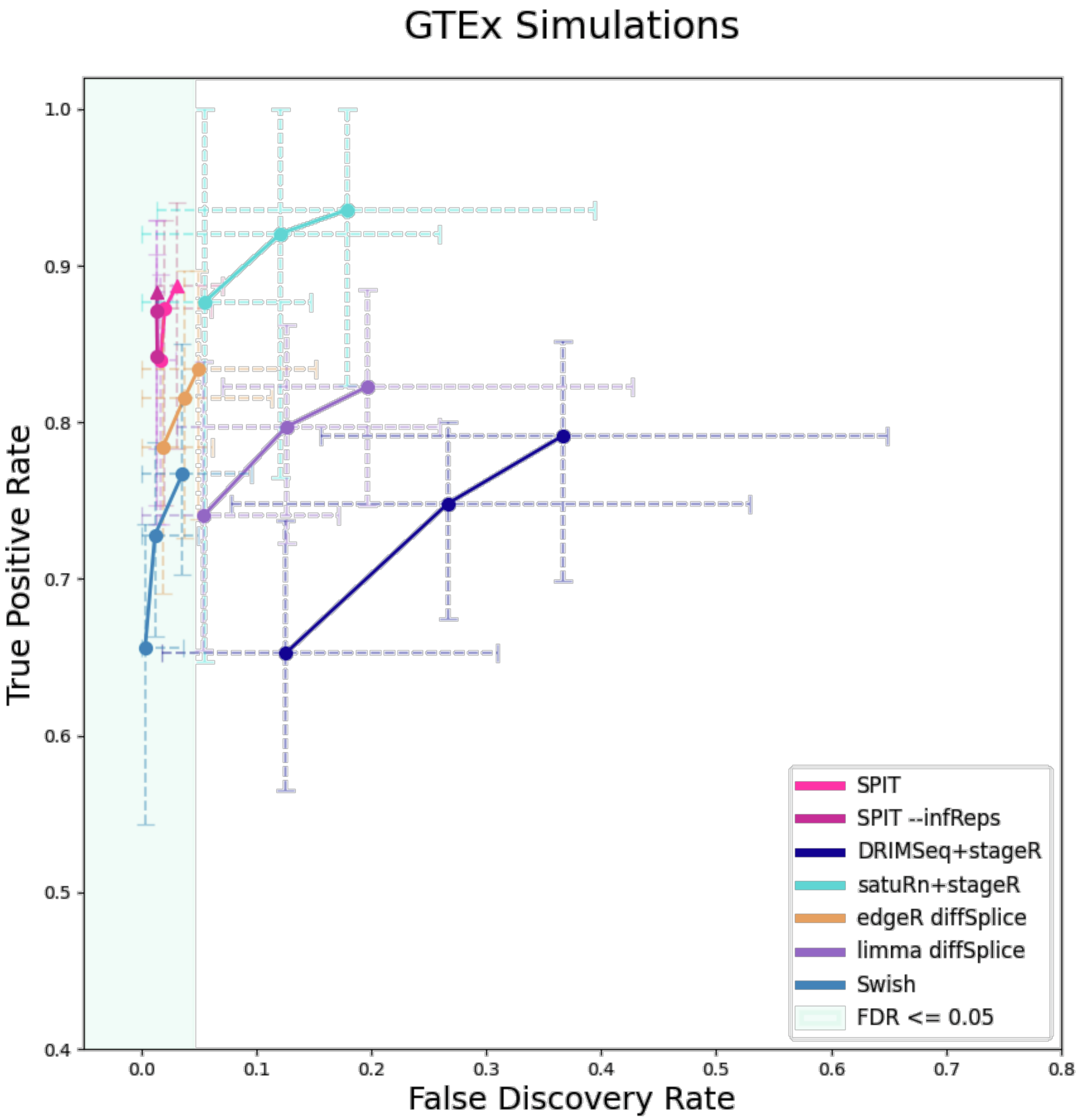

Supplementary Figure 1: Evaluation of DTU methods on GTEx experiments with error bars included for all values of target FDRs and κ for SPIT.

Supplementary Figure 2: Clustering of spliceotypes in experiments 2-19, related to Figure 2

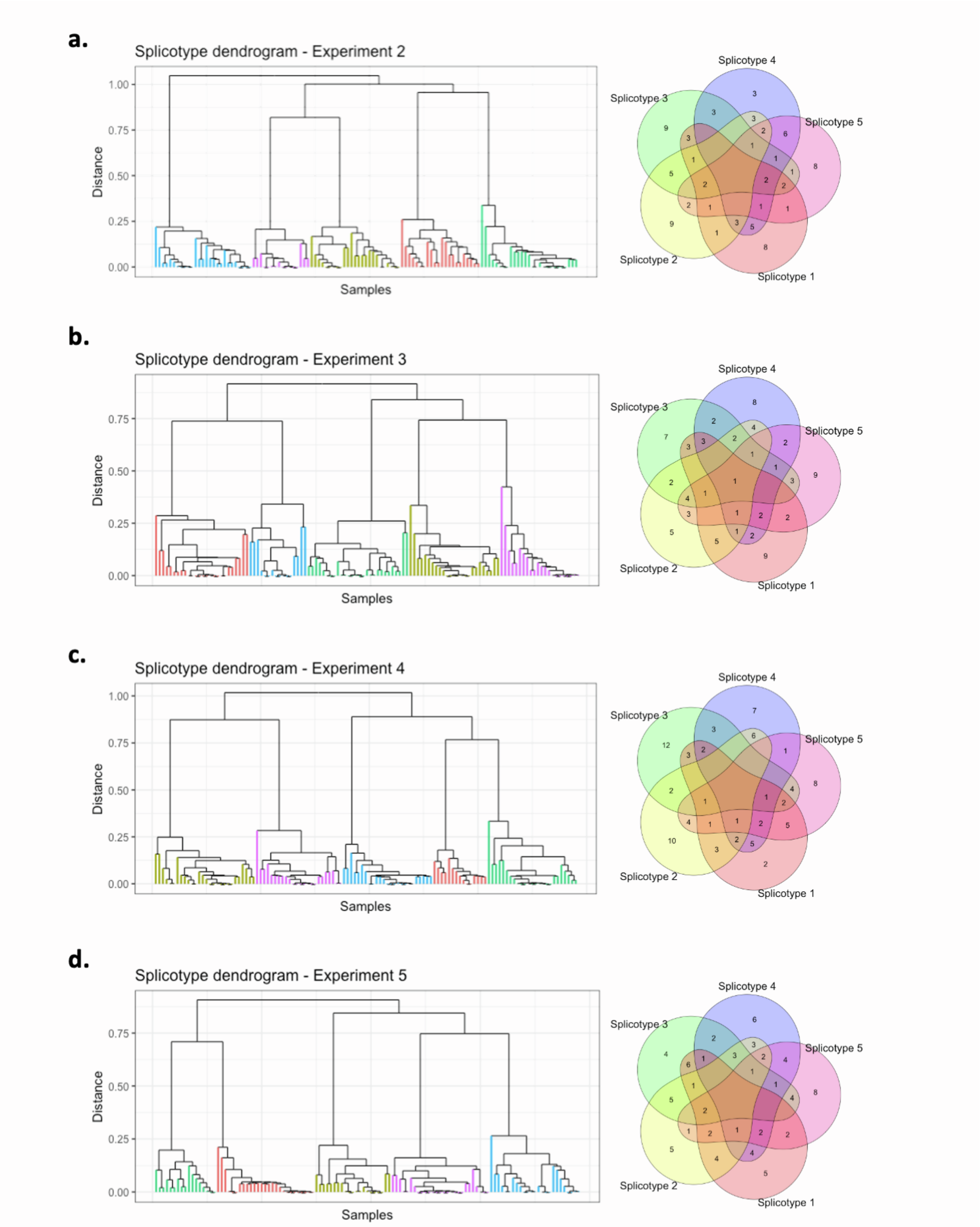

Supplementary Figure 2

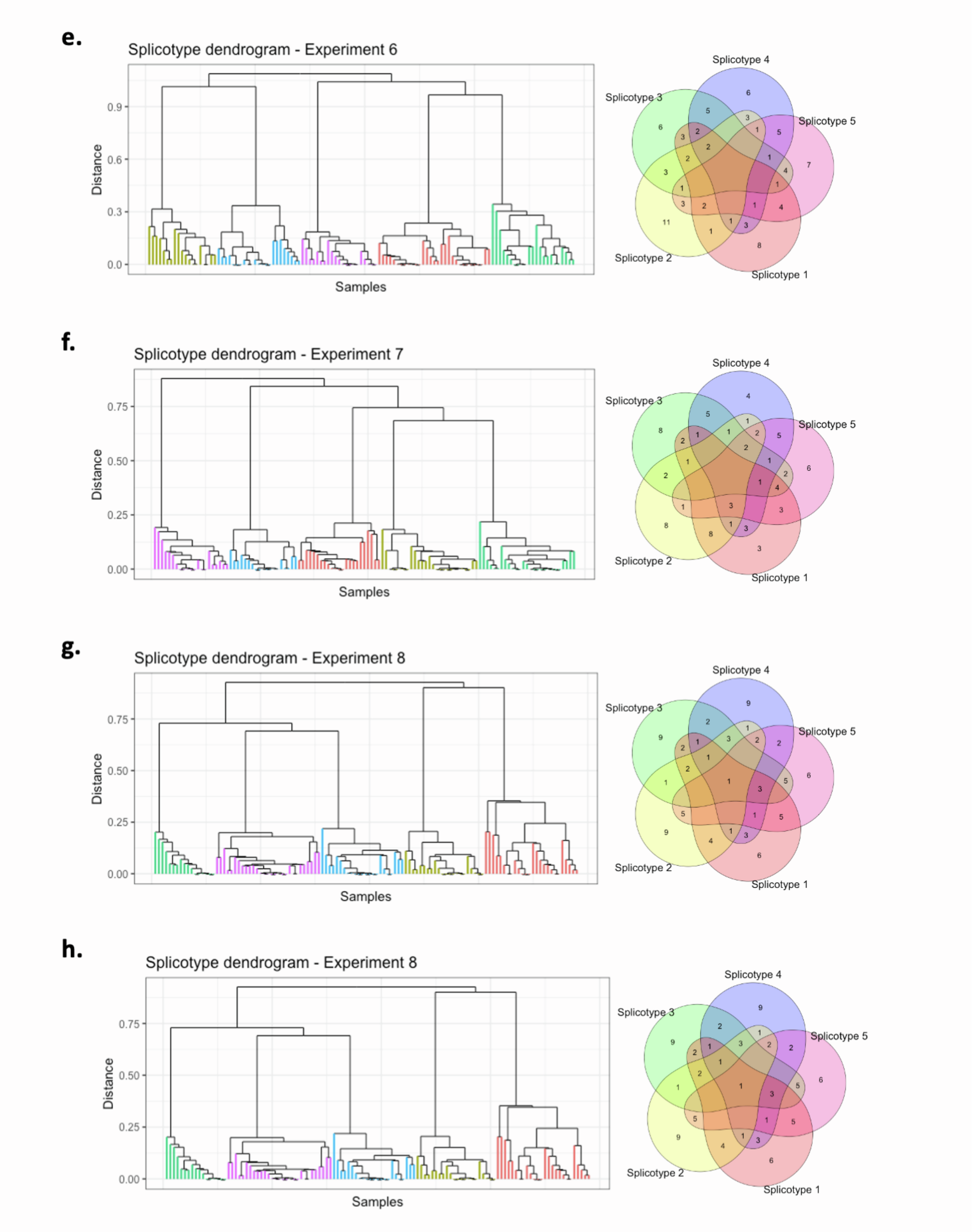

Supplementary Figure 2

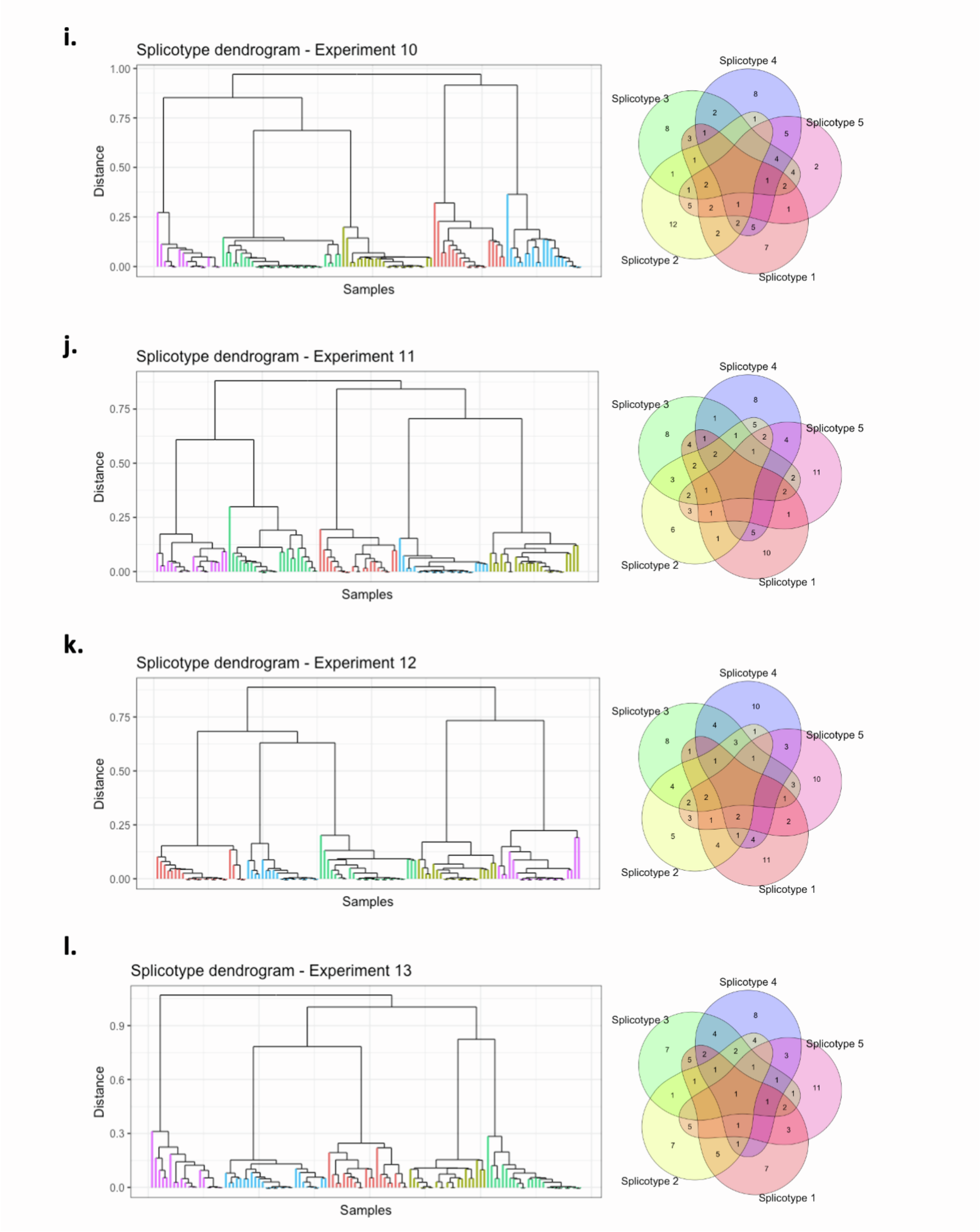

Supplementary Figure 2

m.

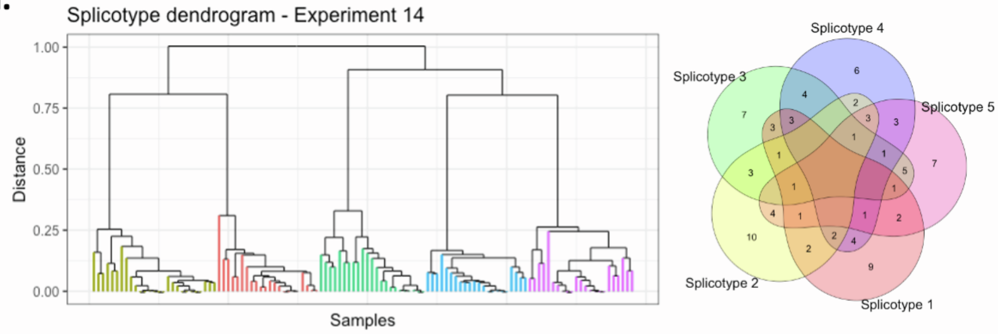

n.

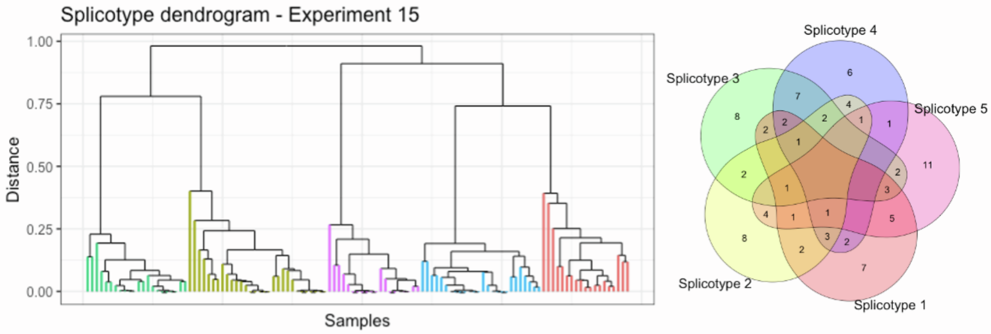

o.

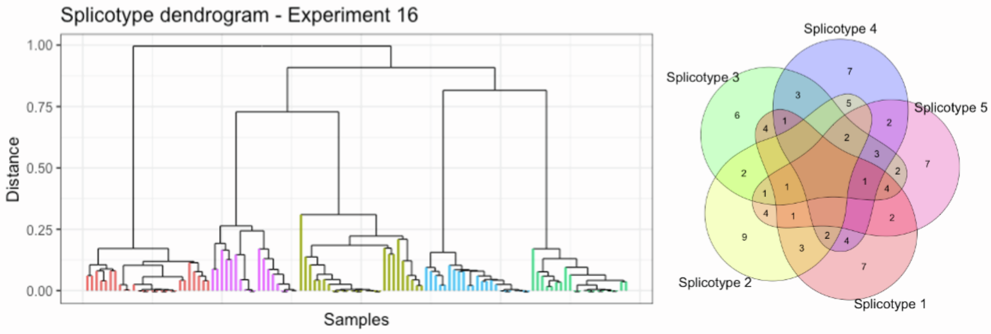

p.

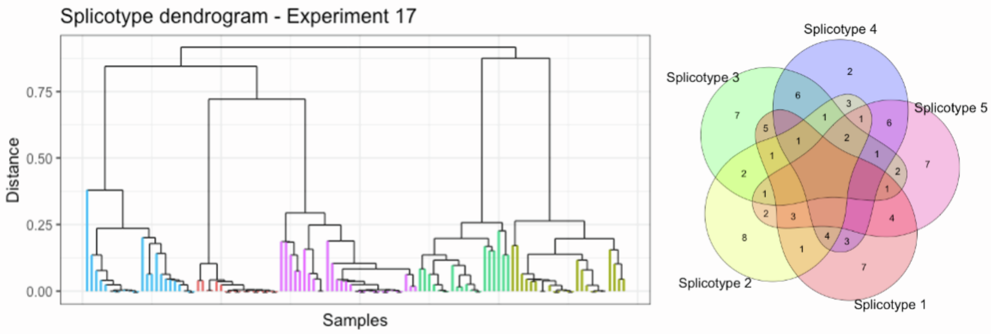

Supplementary Figure 2

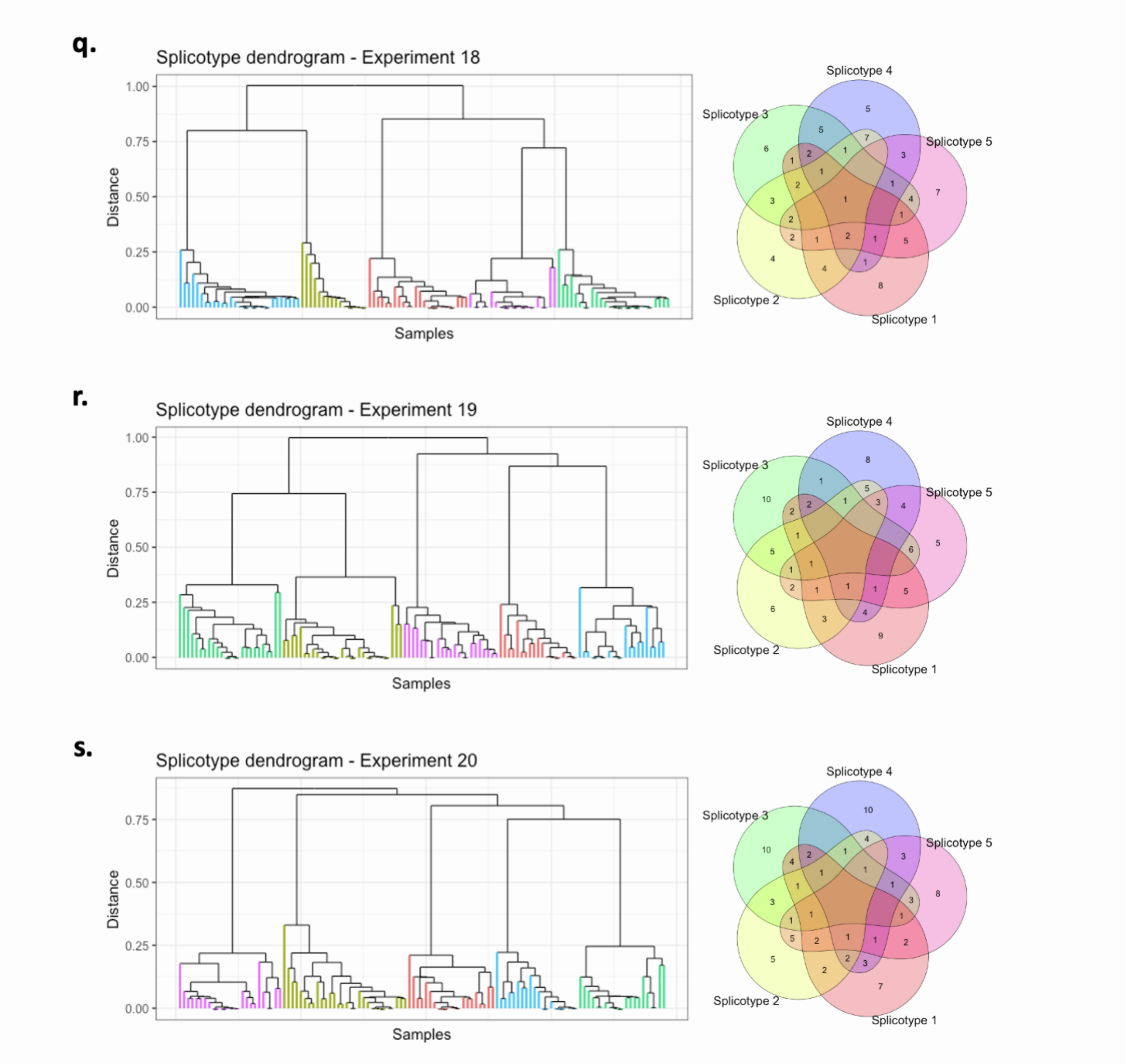

Supplementary Figure 2: The DTU event sharing Venn diagram and the corresponding final subcluster dendrogram based on the SPIT DTU matrix for experiments 2-19. The subclusters are color coded based on their distinct sets of simulated DTU events (spliceotypes).

Supplementary Figure 3: Evaluation with 20 vs. 20 satuRn GTEx simulation, related to Figure 2

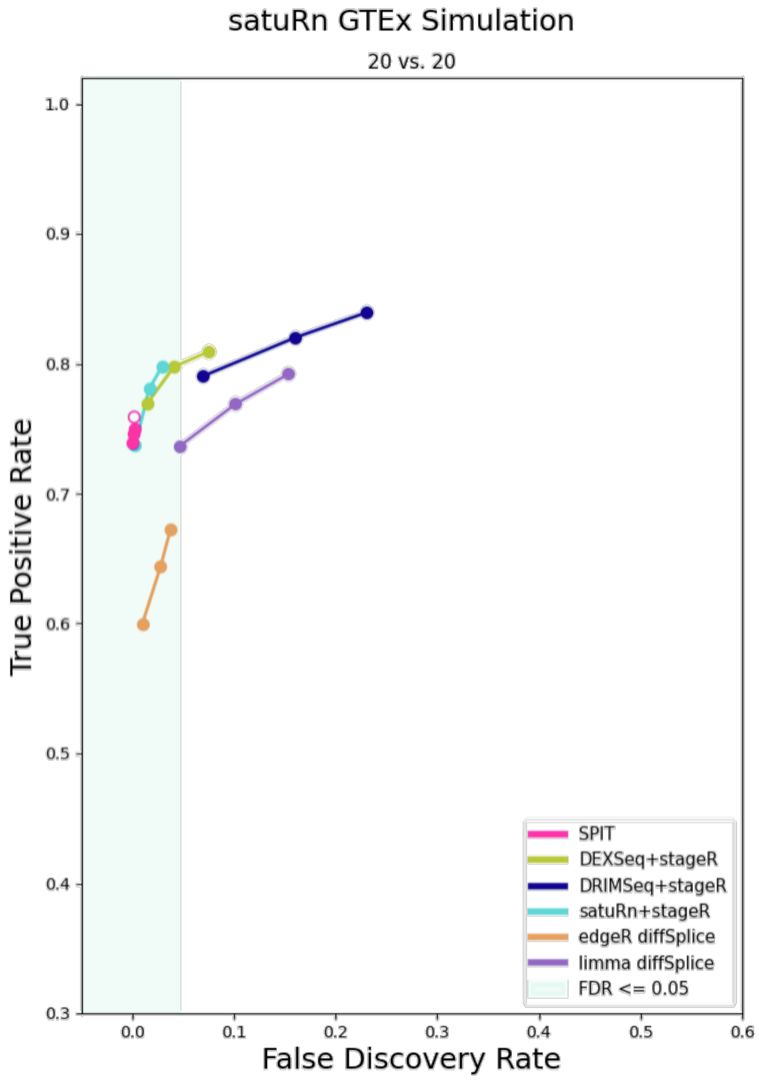

Supplementary Figure 3 : Evaluation of DTU methods on the satuRn GTEx simulation with sample sizes 20 vs. 20.

Supplementary Figure 4: SPIT pre-filtering on Lieber samples, related to STAR Methods and Figure 4

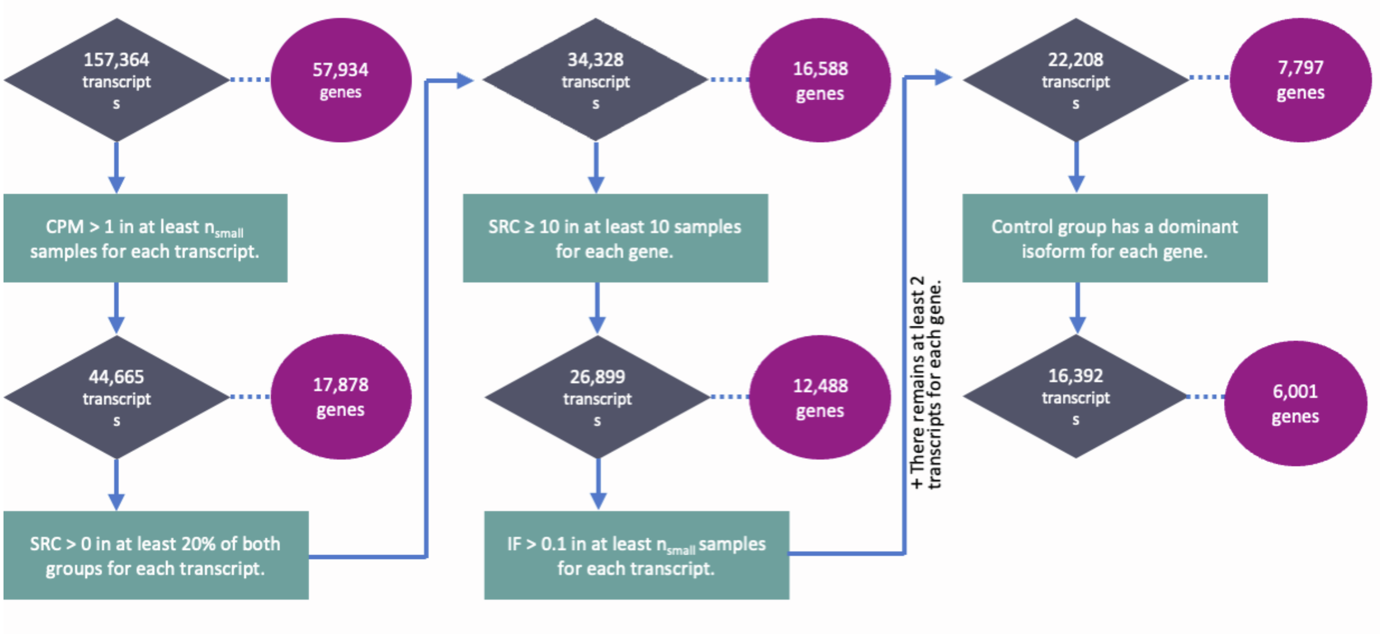

Supplementary Figure 4: Numbers of remaining transcripts and genes after each step of SPIT's pre-filtering process applied on the Lieber brain samples with the default parameters (n_{small} = 12).

Supplementary Figure 5: Pre-filtering investigation, related to STAR Methods and Figure 2

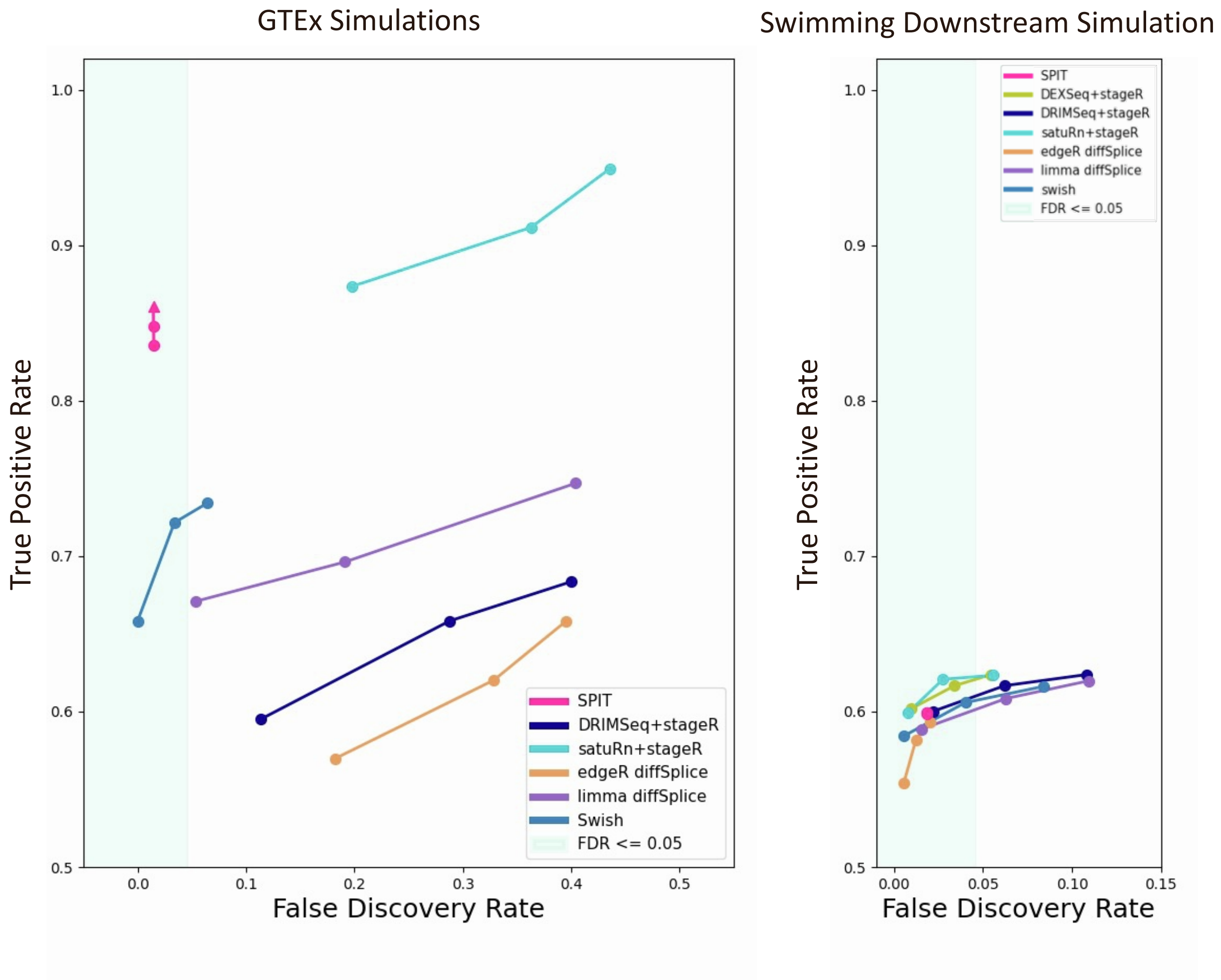

Supplementary Figure 5: Evaluation of DTU methods on GTEx experiments and the Swimming Downstream dataset with switched prefiltering steps: the SPIT prefilters are applied on the Swimming Downstream dataset and the *DRIMSeq* filters are applied on the GTEx experiments.

Supplementary Figure 6: IF distributions in DTU events associated with Schizophrenia, related to Figure 4

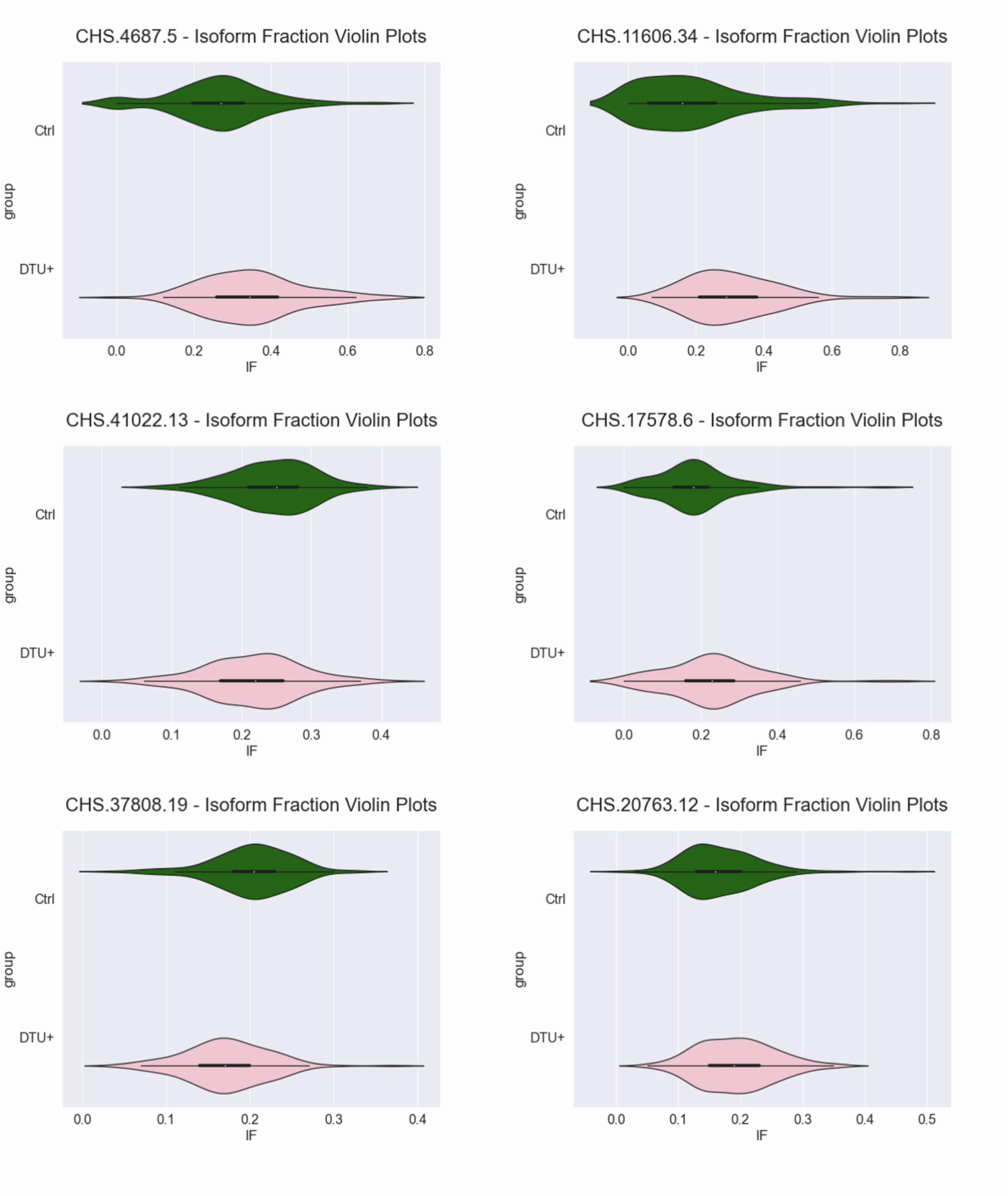

Supplementary Figure 6: Violin plots of the isoform fractions for the 6 transcripts of candidate DTU events in schizophrenia analysis.

Supplementary Table 1: Computation time of each DTU tool on evaluation data sets, related to Figure 2

	DEXSeq	*DRIMSeq*	*satuRn*	*edgeR diffsplice*	*limma diffsplice*	*Swish*	*SPIT*	*SPIT infReps*
**Swimming Downstream (12 vs. 12)**	2m10s	9m17s	0m12s	0m3s	0m2s	1m24s	2m9s	13m30s
**GTEx experiments (117 vs. 118)**	--	41m6s	0m12s	0m23s	0m3s	0m31s	5m10s	34m27s
**satuRn GTEx (20 vs. 20)**	26m49s	32m42s	1m3s	0m15s	0m7s	--	5m16s	--
**satuRn GTEx (50 vs. 50)**	--	51m50s	1m16s	0m31s	0m7s	--	6m33s	--
**Tissue-dependent DTU in SLC25A (497 vs. 380)**	--	2h31m45s	0m36s	1m16s	0m17s	--	33m51s	--

Supplementary Table 2: The total number of samples from the GTEx dataset combined into each tissue, related to Figure 3

Tissue	Number of Samples
Fallopian_Tube	7
Colon	380
Bone_Marrow	102
Uterus	96
Heart	497
Bladder	11
Vagina	101
Lung	380
Blood	616
Ovary	112
Salivary_Gland	70
Blood_Vessel	756
Muscle	480
Brain	1448
Adipose_Tissue	620
Esophagus	803
Breast	220
Prostate	121
Skin	982
Liver	138
Thyroid	361
Kidney	36
Pancreas	199
Small_Intestine	104
Stomach	208
Spleen	118
Cervix_Uteri	11
Nerve	339
Testis	207
Pituitary	130
Adrenal_Gland	161

Supplementary Table 3: Number of candidate DTU genes detected by DTU tools on the GTEx tissue comparisons, related to Figure 3

	DRIMSeq	*satuRn*	*edgeR diffsplice*	*limma diffsplice*	*SPIT*
SLC25A3	3947	420	3343	3777	3349
ANK3	5066	5043	4732	5002	4730
MEF2C	5109	5051	4778	5024	4769
MYO1C	3698	487	3267	3551	3292
